# Supplementary material for: High-throughput quantum photonic devices emitting indistinguishable photons in the telecom C-band
Source: Nat Commun. 2024 Apr 18;15:3358. doi: 10.1038/s41467-024-47551-7 (PMC11026509; doi:10.1038/s41467-024-47551-7)
Supplement: Supplementary file 1 — Supplementary Information [file 41467_2024_47551_MOESM1_ESM.pdf]

Supplementary Information

—

High-throughput quantum photonic devices emitting indistinguishable photons in the telecom C-band

Paweł Holewa,[1,2,3,*] Daniel A. Vajner,[4] Emilia Zięba-Ostój,[1] Maja Wasiluk,[1] Benedek Gaál,[2] Aurimas Sakanas,[2] Marek Burakowski,[1] Paweł Mrowiński,[1] Bartosz Krajnik,[1] Meng Xiong,[2,3] Kresten Yvind,[2,3] Niels Gregersen,[2] Anna Musiał,[1] Alexander Huck,[5] Tobias Heindel,[4] Marcin Syperek,[1,†] and Elizaveta Semenova[2,3,‡]

[1]*Department of Experimental Physics, Faculty of Fundamental Problems of Technology, Wrocław University of Science and Technology, Wyb. Wyspiańskiego 27, 50-370 Wrocław, Poland*
[2]*DTU Electro, Department of Electrical and Photonics Engineering, Technical University of Denmark, Ørsteds Plads 343, DK-2800 Kongens Lyngby, Denmark*
[3]*NanoPhoton - Center for Nanophotonics, Technical University of Denmark, Ørsteds Plads 345A, DK-2800 Kongens Lyngby, Denmark*
[4]*Institute of Solid State Physics, Technische Universität Berlin, 10623 Berlin, Germany*
[5]*Center for Macroscopic Quantum States (bigQ), Department of Physics, Technical University of Denmark, DK-2800 Kongens Lyngby, Denmark*

CONTENTS

Supplementary Note 1. Optical simulations of the cavity geometry 2

Supplementary Note 2. Nanofabrication of the devices 3
A. MOVPE growth of InAs/InP quantum dots 3
B. Deterministic fabrication of the cavities 3

Supplementary Note 3. Accuracy of determination of QD positions from µPL maps and final cavity positioning 4
A. Signal-to-noise ratio 4
B. Localization algorithm 5
C. Uncertainty of scaling the µPL maps 5
D. Image rotation φ 6
E. One-dimensional QD position ΔQ_i 7
F. Two-dimensional QD position ΔQ 7
G. Distance between QD position and cavity center R – accuracy of cavity positioning ΔR 7
H. Example calculations for QD-CBGs #1–#3 7
I. Calculation of the diffraction-limited spot size 7
J. Statistics on QD spot size and the accuracy of QD positioning 8

Supplementary Note 4. Optical setups 9

Supplementary Note 5. Determination of the photon extraction efficiency 9

Supplementary Note 6. Supporting microphotoluminescence data for QD-CBG devices 11
A. Quantum dot linewidths 11
B. Time-resolved microphotoluminescence 11
C. Temperature-dependent µPL 12

Supplementary Note 7. Quantum optics experiments 13
A. Off-resonant autocorrelation data for QD-CBG #2 13
B. Microphotoluminescence excitation spectroscopy of QD-CBG #2 14
C. Quasi-resonant autocorrelation data for QD-CBG #2 15
D. Indistinguishability measurements and data analysis 15

* pawel.holewa@pwr.edu.pl
† marcin.syperek@pwr.edu.pl
‡ esem@fotonik.dtu.dk

E. Coherence measurements 19

Supplementary References 23

Supplementary Note 1. OPTICAL SIMULATIONS OF THE CAVITY GEOMETRY

The CBG geometry is modeled using a modal method employing a true open geometry boundary condition [1]. Here, the geometry is divided into uniform layers along a propagation z axis, and the field is expanded in eigenmodes of each uniform layer. The QD is modeled as a classical dipole emitter using the equivalence principle [2]. The eigenmode expansion coefficients in the QD layer are computed using the reciprocity theorem [3], and the fields are connected at each layer interface using the S matrix formalism [3, 4].

We model the Purcell factor $F_P = P/P_0$ as the power P emitted by the classical dipole relative to the power P_0 in a bulk medium. The power P and the electric near field $\mathbf{E}(\mathbf{r})$ generated by a dipole $\mathbf{d}$ with frequency ω_0 at the position $\mathbf{r}_0$ can be written in terms of the optical Green's function $\overleftrightarrow{\mathbf{G}}(\mathbf{r},\mathbf{r}')$ as [2]

$$P(\mathbf{r}_0) = \frac{\omega_0^3 \mu_0 |\mathbf{d}|^2}{2} \mathrm{Im}\left(\mathbf{n}_\mathrm{d}^* \cdot \overleftrightarrow{\mathbf{G}}(\mathbf{r}_0,\mathbf{r}_0) \cdot \mathbf{n}_\mathrm{d}\right) \tag{1}$$

$$\mathbf{E}(\mathbf{r}) = \omega_0^2 \mu_0 \overleftrightarrow{\mathbf{G}}(\mathbf{r},\mathbf{r}_0)\mathbf{d}, \tag{2}$$

where $\mathbf{n}_\mathrm{d} = \mathbf{d}/|\mathbf{d}|$ is the dipole orientation. The corresponding far field $P_\mathrm{FF}(\theta,\varphi,\mathbf{r}_0)$ is then determined from Supplementary Eq. (2) using a standard near field to far field transformation [5]. The total collected power P_Lens detected by the lens with a given numerical aperture (NA) is obtained by integration of $P_\mathrm{FF}(\theta,\varphi,\mathbf{r}_0)$ over the unit solid angle Ω as

$$P_\mathrm{Lens}(\mathbf{r}_0) = \int_{\theta<\theta_\mathrm{NA}} P_\mathrm{FF}(\theta,\varphi,\mathbf{r}_0)\,\mathrm{d}\Omega, \tag{3}$$

where θ_NA is defined by the NA of the lens. Finally, the extraction efficiency is defined as $\eta = P_\mathrm{Lens}/P$.

The dominant lines in all investigated QD-CBG devices are trions (CX) which emit circularly polarized photons ($\sigma^\pm$). We thus model the trion state dipole orientation as

$$\mathbf{n}_\mathrm{d} = \mathbf{n}_\mathrm{CX} = \frac{1}{\sqrt{2}}(\mathbf{r} \pm i\boldsymbol{\varphi}), \tag{4}$$

where $\mathbf{r}$ and $\boldsymbol{\varphi}$ are unit vectors of the cylindrical coordinate system. Inserting Supplementary Eq. (4) into Supplementary Eq. (1), we obtain the power P_CX emitted by the trion given by

$$P_\mathrm{CX}(\mathbf{r}_0) = \frac{P_r(\mathbf{r}_0) + P_\varphi(\mathbf{r}_0)}{2}, \tag{5}$$

where P_r (P_φ) is the power emitted by a dipole at position $\mathbf{r}_0$ oriented along the r (φ) axis. Similarly, the far field generated by the trion becomes

$$P_\mathrm{FF,CX}(\theta,\varphi,\mathbf{r}_0) = \frac{1}{2}\left(P_{\mathrm{FF},r}(\theta,\varphi,\mathbf{r}_0) + P_{\mathrm{FF},\varphi}(\theta,\varphi,\mathbf{r}_0)\right), \tag{6}$$

where r and φ again refer to far fields generated by the two dipole orientations. Finally, the total photon extraction efficiency for the trion at the position $\mathbf{r}_0$ becomes

$$\eta_\mathrm{CX}(\mathbf{r}_0) = \frac{P_\mathrm{Lens,CX}(\mathbf{r}_0)}{P_\mathrm{CX}(\mathbf{r}_0)} = \frac{P_{\mathrm{Lens},r}(\mathbf{r}_0) + P_{\mathrm{Lens},\varphi}(\mathbf{r}_0)}{P_r(\mathbf{r}_0) + P_\varphi(\mathbf{r}_0)}. \tag{7}$$

The near and far fields for the cavity mode are presented in Supplementary Fig. 1. The near-field profile shown in Supplementary Fig. 1a evidences the higher-order nature of the optimized cavity mode characteristic of the CBG design [6].

The extraction efficiency η and Purcell factor F_P computed using Supplementary Eq. (5) and Supplementary Eq. (7) as a function of spatial misalignment ρ of the QD is presented in Supplementary Fig. 2. Whereas the photon extraction efficiency overall displays robustness towards misalignment, the decay of the Purcell factor with ρ is much more pronounced. The variations of F_P along the r and φ axes are quite different and result from the different variations of the field profile shown in Supplementary Fig. 1 along the x and y axes.

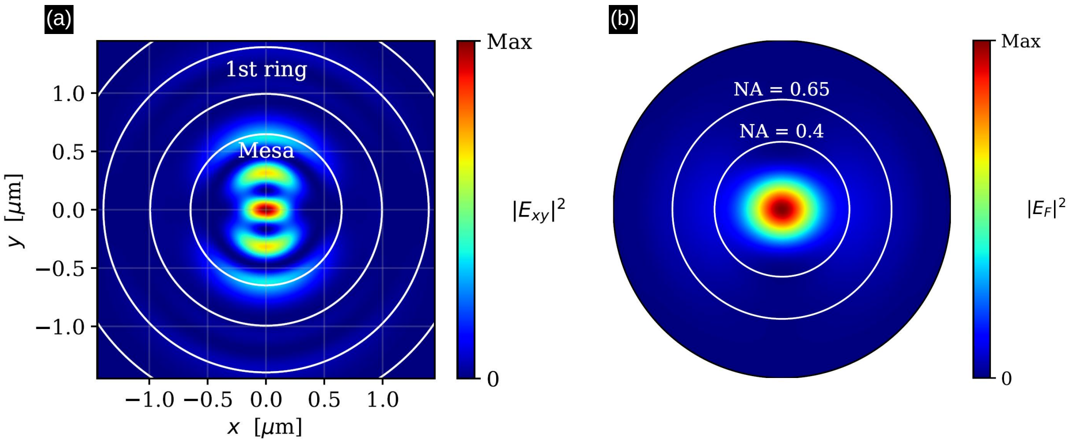

Supplementary Fig. 1. Circular Bragg grating cavity mode. **a**, Near field and **b**, far-field mode profiles of the CBG with 4 rings generated by a linear dipole $\mathbf{n}_\mathrm{d} = \mathbf{r}$. E_F – amplitude of electric field, E_{XY} – amplitude of the in-plane electric field.

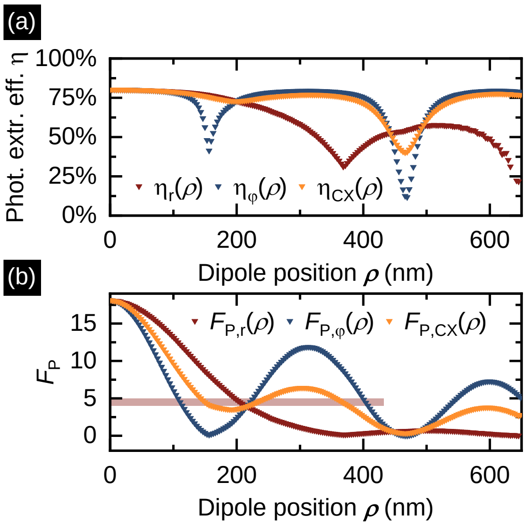

Supplementary Fig. 2. Quantum dot displacement tolerance. **a**, the photon extraction efficiency $\eta(\rho)$ and **b**, Purcell factor $F_\mathrm{P}(\rho)$ as a function of the dipole-center separation ρ computed for the r, φ and trion (CX) dipole orientations.

Supplementary Note 2. NANOFABRICATION OF THE DEVICES

A. MOVPE growth of InAs/InP quantum dots

Supplementary Figure 3 presents the atomic force microscopy (AFM) image of a reference structure that has the same InAs/InP quantum dots (grown under nominally same conditions) without InP capping layer. We estimate the density of QDs to be $3.1 \times 10^8/\mathrm{cm}^2$ at the center of the wafer where the imaging fields are fabricated.

B. Deterministic fabrication of the cavities

Supplementary Fig. 4 shows the optical microscope image of the sample's surface taken at 20× magnification with the cavities transferred to InP. The imaged fragment of the chip shows a 5×5 pattern of the imaging fields with four InP crosses at the corners of the pattern. Also visible are additional, 2 μm side-length square mesas.

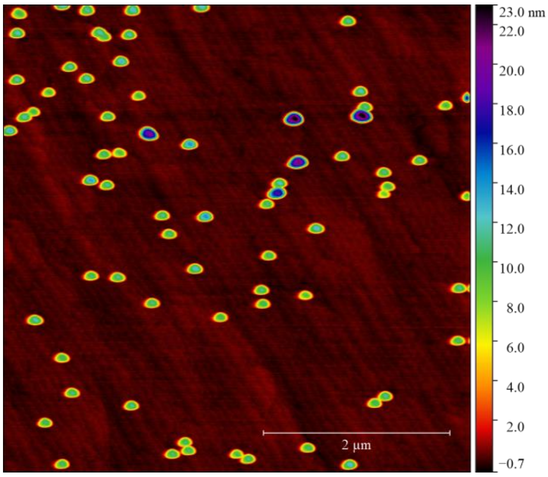

Supplementary Fig. 3. Atomic force microscopy (AFM) image of the InP surface with InAs quantum dots grown under nominally the same conditions as those used for the imaging.

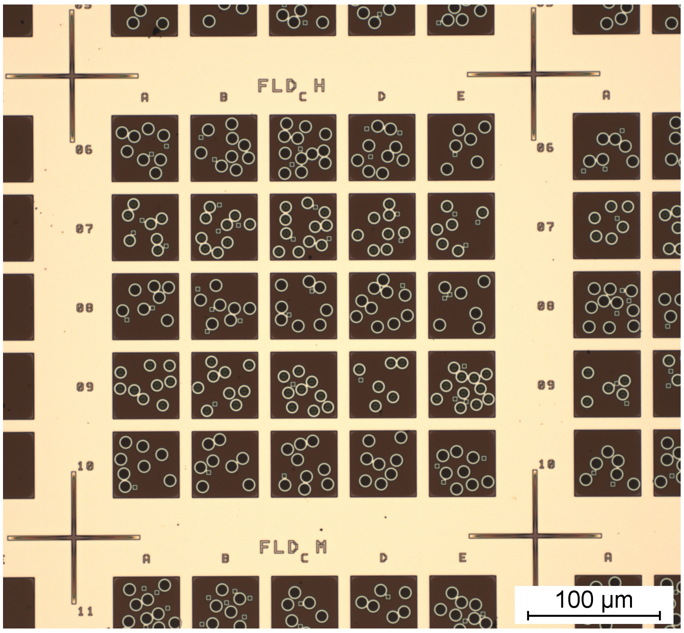

Supplementary Fig. 4. The optical microscope image of the sample's surface taken at 20× magnification with the cavities transferred to InP.

Supplementary Note 3. ACCURACY OF DETERMINATION OF QD POSITIONS FROM µPL MAPS AND FINAL CAVITY POSITIONING

A. Signal-to-noise ratio

Supplementary Fig. 5 shows the histogram of signal-to-noise ratio (SNR) for the mapped QDs. The average SNR is 10.6 which underlines the crucial role of the 7-fold enhancement of the signal intensity for the planar structure by application of the metallic mirror [7]. Based on this result, we deduce that for a sample without such a mirror, the SNR would be roughly 7 times lower (SNR of about 1.5), which would make the optical localization of QDs impossible (compare with the noise level in Figs. 2e and 2f in the main text).

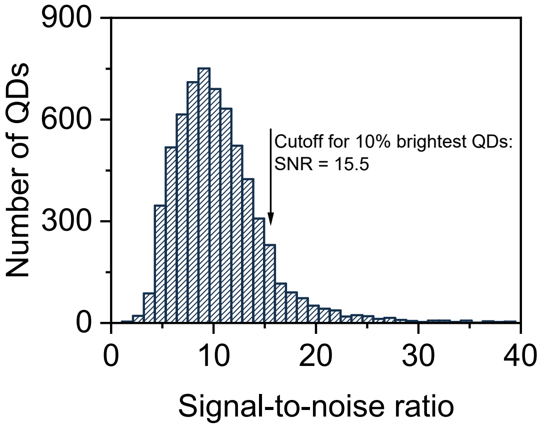

Supplementary Fig. 5. Histogram of signal-to-noise ratio (SNR) for the recorded QD spots.

B. Localization algorithm

Our algorithm calculates the scaling factor P [px/µm] to change the map unit from pixels (px) to µm. For a given µPL map, we identify n QD spots and take $2n$ cross-sections of the map (each QD is sectioned in two directions). The width of each cross-section is taken to equal a few pixels centered at the QD spot maximum to increase the signal-to-noise ratio (SNR). We found the best SNR for 10 pixels and used this value consistently.

For the i-th cross-section, we determine the positions $\mathbb{M}_{i,l}$ [px], $\mathbb{M}_{i,u}$ [px][1] of the field boundaries (acting as reference/alignment marks, AMs; indices l and u stand for lower and upper field boundaries, the same indices are applied also for left and right field edges) and $\mathbb{Q}_i$ [px] for the QD spot position. All values $\mathbb{M}_{i,l}$, $\mathbb{M}_{i,u}$, and $\mathbb{Q}_i$ are the centers of the Gaussian peaks, fitted to respective maxima on the µPL map cross-section. Additionally, we assume that each field is a square of size $F = 50\,\mu$m.

P is calculated by averaging over all cross-section-related coefficients P_i recorded for a given field ($P = \bar{P}_i$) to ensure its highest accuracy, according to the formula

$$P = \frac{1}{2n}\sum_{i=1}^{2n} P_i = \frac{1}{2n}\sum_{i=1}^{2n} \frac{|\mathbb{M}_{i,u} - \mathbb{M}_{i,l}|}{F}. \tag{8}$$

Calculating P separately for each map accounts for possible slight changes in the magnification due to defocusing of the sample surface during the cryostat translation, however, we find very low dispersion of P coefficients for different µPL maps (see the following section and Supplementary Fig. 6).

Then, the i-th QD position Q_i [µm] (vertical or horizontal) is calculated as

$$Q_i = \frac{\mathbb{Q}_i - \mathbb{M}_{i,l}}{P}. \tag{9}$$

C. Uncertainty of scaling the µPL maps

The accuracy of the scaling factor ΔP_i for a single (i-th) µPL map cross-section can be calculated by propagating the uncertainties in Supplementary Eq. (8):

$$\Delta P_i = \sqrt{\left(\frac{\Delta\mathbb{M}_{i,u}}{F}\right)^2 + \left(\frac{\Delta\mathbb{M}_{i,l}}{F}\right)^2 + \left(\frac{\Delta F}{F^2}\right)^2}.$$

The uncertainty of the field size ΔF has two contributions, the uncertainty of the electron beam lithography alignment, estimated to $\Delta C = 40\,$nm [8] and the over-etching Δx during the ICP-RIE step (estimated to be up to $\Delta x = 40\,$nm), potentially influencing F by $2\Delta x = 80\,$nm. $\Delta\mathbb{M}_{i,u}$ and $\Delta\mathbb{M}_{i,l}$ are standard errors of the numerical fitting.

---

[1] For clarity, we use the blackboard-bold font for quantities given in pixels, e. g. $\mathbb{M}$, $\mathbb{Q}$. The normal font is used for the same dimensions given in µm, e. g. Q.

However, to increase the accuracy of the determination of the scaling factor, we average the P_i values for all cross-sections taken in a particular µPL map, as can be seen in Supplementary Eq. (8), so as the uncertainty ΔP we adopt the estimator of the standard error of mean $\hat{\sigma}_{\bar{P}_i}$ for the sample defined as a set of all $2n$ factors $P_i = |\mathbb{M}_{i,u} - \mathbb{M}_{i,l}|/F$ determined for a given µPL map:

$$\Delta P = \hat{\sigma}_{\bar{P}_i} = \frac{\sigma_{P_i}}{\sqrt{2n}}, \tag{10}$$

where σ_{P_i} is the sample standard deviation.

According to calculated statistics for $m = 84$ exemplary µPL maps, we can compare the estimators of the standard error of mean $\hat{\sigma}_{\bar{P}_i}$ determined separately for each µPL map and reach the following conclusions:

1. We find that the coefficients P_i are very close to each other, evidencing no perceptible elongation or distortion of the registered images. This can be evaluated by the analysis of the set of calculated (for all m maps) uncertainties $\Delta P = \hat{\sigma}_{\bar{P}_i}$ scaled by the determined P factors: $\delta P = \Delta P/P$. We plot the histogram of δP in Supplementary Fig. 6 and find the median value of δP-distribution of 0.06 %. In other words, for half of the analyzed maps, the distribution of P_i factors is sufficiently narrow to determine the P factor with relative uncertainty $\delta P < 0.06\,\%$. This value includes the potential elongation of the image (difference in vertically and horizontally determined P_i factors).

2. The stability of the setup and overall repetitivity of the imaging process is high as the image magnification varies very little between maps. This can be evaluated by the estimator of the standard error of mean $\hat{\sigma}_{\bar{P}}$ calculated not for different cross-sections [averaging P_i values for a given map, as in Supplementary Eq. (8)] but for different maps (averaging P values for all maps). We obtain $\hat{\sigma}_{\bar{P}}/\bar{P} = 0.013\,\%$. We take this value as the estimation of variation of the setup magnification during the imaging process.

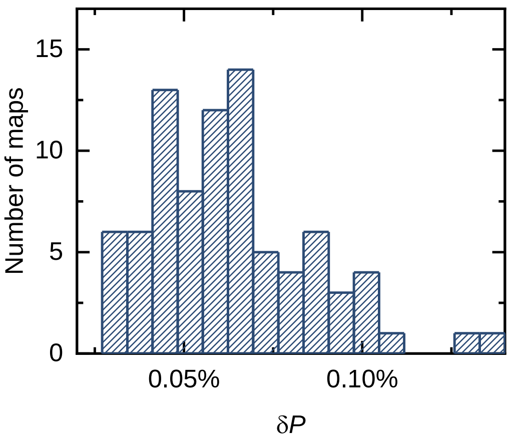

Supplementary Fig. 6. The histogram of relative microphotoluminescence map scaling uncertainty $\delta P = \Delta P/P$ for the set of analyzed $m = 84$ exemplary microphotoluminescence maps.

Then, the accuracy of cavity positioning ΔR depends primarily on the accuracy of the QD localization (ΔQ) and EBL alignment uncertainty ΔC. The ΔQ itself depends on the fit standard errors ($\Delta\mathbb{M}$, $\Delta\mathbb{Q}$), and scaling factor uncertainty ΔP.

D. Image rotation φ

Although we carefully align the image of the field with the horizontal and vertical axes of the detector array, we assume that there can be some indiscernible rotation of the image φ on the level of up to a few degrees. Even if this is the case, the rotation results first in the larger separation between AM peaks in the cross-sections ($|\mathbb{M}_{i,u} - \mathbb{M}_{i,l}|/\cos\varphi$ instead of $|\mathbb{M}_{i,u} - \mathbb{M}_{i,l}|$) and this is translated to scaled P: $P \to P/\cos\varphi$, according to Supplementary Eq. (8). The distance between lower AM and QD is however also elongated, $\left(\mathbb{Q}_i - \mathbb{M}_{i,l}\right)/\cos\varphi$ instead of $\left(\mathbb{Q}_i - \mathbb{M}_{i,l}\right)$. According to Supplementary Eq. (9), the new QD position is:

$$Q_{i,\text{new}} = \frac{\left(\mathbb{Q}_i - \mathbb{M}_{i,l}\right)/\cos\varphi}{P/\cos\varphi} = Q_i,$$

so that the slight image rotation has no influence on the determination of QD position Q_i in our approach.

E. One-dimensional QD position ΔQ_i

The determination of i-th QD position Q_i is influenced by the scaling factor uncertainty ΔP, as well as QD ($\Delta\mathbb{Q}_i$) and lower AM ($\Delta\mathbb{M}_{i,l}$) fit uncertainties (standard errors of the numerical fitting), and is calculated by propagating the uncertainties, according to Supplementary Eq. (9):

$$\Delta Q_i = \sqrt{\left(\frac{\Delta\mathbb{Q}_i}{P}\right)^2 + \left(\frac{\Delta\mathbb{M}_{i,l}}{P}\right)^2 + \left(\frac{\left(\mathbb{Q}_i - \mathbb{M}_{i,l}\right)\Delta P}{P^2}\right)^2}. \quad (11)$$

F. Two-dimensional QD position ΔQ

We combine the one-dimensional QD position uncertainties ΔQ_i into the two-dimensional uncertainty using the formula

$$\Delta Q = \sqrt{(\Delta Q_h)^2 + (\Delta Q_v)^2}, \quad (12)$$

where ΔQ_h, ΔQ_v are ΔQ_i values calculated according to Supplementary Eq. (11) for horizontal and vertical cross-sections. We use the ΔQ value to determine the accuracy of our µPL imaging method.

G. Distance between QD position and cavity center R – accuracy of cavity positioning ΔR

We express the uncertainty ΔR of the expected distance $R = 0$ between the QD position and the cavity center as

$$\Delta R = \sqrt{(\Delta Q)^2 + (\Delta C)^2}. \quad (13)$$

H. Example calculations for QD-CBGs #1–#3

Finally, in Supplementary Table 1 we show the uncertainties involved in the determination of the QD position ΔQ_i accordingly to Supplementary Eq. (11) and of the accuracy of cavity positioning ΔR accordingly to Supplementary Eq. (13) for three exemplary QD-CBGs #1–#3, described in the article (QD-CBG #1 and QD-CBG #2) and in the following part of this document (QD-CBG #3).

Supplementary Table 1. Uncertainties involved in the determination of accuracy of cavity positioning ΔR for exemplary quantum dot-circular Bragg grating (QD-CBGs) devices #1–#3. $\Delta\mathbb{Q}_i/P$ – 1D fit uncertainty for the QD peak center determination, $\Delta\mathbb{M}_i/P$ – 1D fit uncertainty for the alignment mark (AM) center determination, ΔQ_i – 1D uncertainty of QD localization, ΔQ – uncertainty of QD localization in 2D.

Device	Orientation	$\Delta\mathbb{Q}_i/P$	$\Delta\mathbb{M}_i/P$	$\left(\mathbb{Q}_i - \mathbb{M}_{i,l}\right)\Delta P/P^2$	ΔQ_i	ΔQ	ΔR
QD-CBG #1	Vertical	61.0 nm	14.4 nm	19.9 nm	65.8 nm	141.9 nm	147.4 nm
	Horizontal	120.0 nm	29.0 nm	23.5 nm	125.7 nm		
QD-CBG #2	Vertical	61.9 nm	12.7 nm	20.1 nm	66.3 nm	137.3 nm	143.0 nm
	Horizontal	115.7 nm	26.1 nm	20.2 nm	120.2 nm		
QD-CBG #3	Vertical	112.2 nm	20.7 nm	12.0 nm	114.7 nm	132.9 nm	138.8 nm
	Horizontal	59.5 nm	19.5 nm	24.6 nm	67.2 nm		

I. Calculation of the diffraction-limited spot size

In this and the following subsections, we consider a QD as a point light source and calculate the expected observed width of such a source in our imaging setup shown in Fig. 2a of the main text. Its emission can be described by the point spread function

(PSF) of the setup, which for the approximation of 2D paraxial wide-field fluorescence microscope forms an Airy disc (first-order Bessel function of the first kind) [9].

Our QD localization algorithm uses the approximation of the Bessel function by the Gaussian profile, which we use for fitting the QD- and AM-related signal in the cross-sections of µPL maps.

First, we calculate the standard deviation σ_{diff} of the Gaussian curve that best approximates the PSF of our setup according to the formula [9]:

$$\sigma_{\text{diff}} \approx 0.21 \frac{\lambda_{\text{QD}}}{\text{NA}} = 0.501\,\mu\text{m} \tag{14}$$

with employed NA = 0.65 and $\lambda_{\text{QD}} = 1.55\,\mu$m. Then, we translate σ_{diff} to full width at half-maximum (FWHM) as

$$\text{FWHM}_{\text{diff}} = 2\sqrt{2\ln 2} \times \sigma_{\text{diff}} \approx 1.18\,\mu\text{m}. \tag{15}$$

J. Statistics on QD spot size and the accuracy of QD positioning

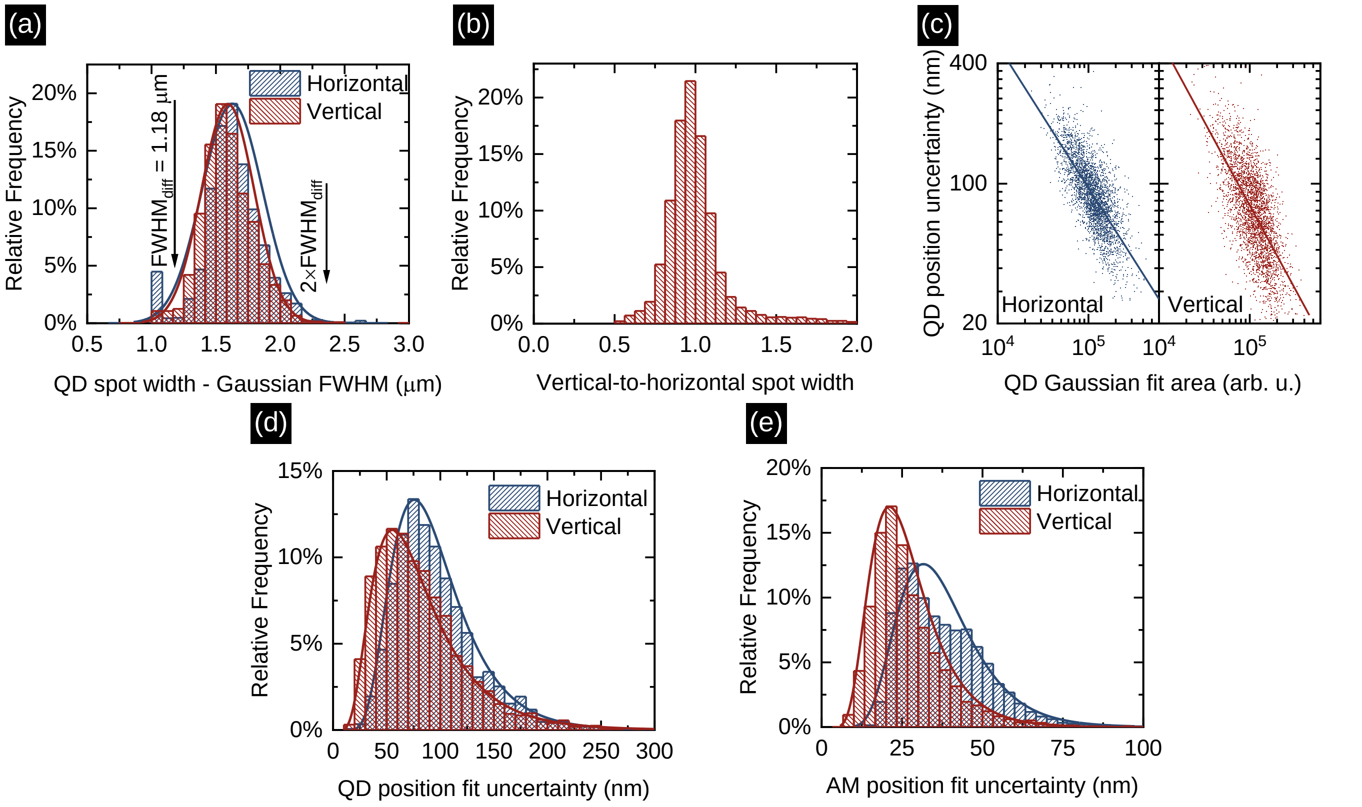

Supplementary Fig. 7. Parameters of fitting the microphotoluminescence map cross-sections, shown separately for horizontal and vertical directions. **a**, the histogram of full width at half maximum (FWHM) values for the fits used for the determination of quantum dot (QD) position Q compared with the calculated diffraction-limited value $\text{FWHM}_{\text{diff}} = 1.18\,\mu$m [Supplementary Eq. (15)] and fitted with the normal distribution curves, **b**, histogram of the ratio between the widths of the fitted horizontal and vertical cross-sections of the QD Gaussian profiles, based on the panel (a), **c**, QD position fit uncertainty $\Delta\mathbb{Q}/P$ as a function of the QD fit area, **d**, histogram of the QD position fit uncertainty $\Delta\mathbb{Q}/P$, **e**, alignment mark (AM) position fit uncertainty $\Delta\mathbb{M}/P$. Histograms in panels (d) and (e) are fitted with log-normal distributions.

We take the calculated $\text{FWHM}_{\text{diff}} = 1.18\,\mu$m (see previous section) as the diffraction-limited spot size and compare it with the histogram of all FWHM values for registered QD spots in Supplementary Fig. 7a. The medians for the registered FWHM values are similar for horizontal and vertical cross-sections, $1.62\,\mu$m and $1.58\,\mu$m, respectively, which is $\sim$35 % more than the calculated $\text{FWHM}_{\text{diff}}$. Almost all registered FWHM values are lower than $2\times\text{FWHM}_{\text{diff}}$. Therefore, our imaging setup operates close to the diffraction limit with residual broadening originating most probably from the cryostat window between the QDs and microscope objective [10]. The similarity between horizontal and vertical spot widths is evidenced by the histograms of ratios

between the widths of the fitted horizontal and vertical cross-sections of the QD Gaussian profiles (Vertical-to-horizontal spot width), presented in Supplementary Fig. 7b. The average ratio is determined as 99.78 %, in other words, it differs only by 0.22 % from the ideal case unity ratio. The standard sample deviation for the ratio values distribution is calculated to be $\sigma = 19.24\,\%$.

As expected, the fitting uncertainty $\Delta\mathbb{Q}_i/P$ is correlated with the QD brightness, here estimated by the Gaussian fit area, and the corresponding plot is shown in Supplementary Fig. 7c. We find a strong negative correlation between considered fit parameters (Pearson correlation coefficient of -0.68 and -0.73 for horizontal and vertical cross-sections, respectively).

Supplementary Figure 7d presents the fitting uncertainty for QD positions $\Delta\mathbb{Q}_i/P$, separately for horizontal and vertical cross-sections. The uncertainty is defined as the standard error of the fitting procedure for estimation of the center of the Gaussian profile and in all analyzed cases, the obtained values form positively skewed distributions that follow the log-normal distribution, as shown with fit curves. The medians for $\Delta\mathbb{Q}_i/P$ are 88.6 nm and 73.6 nm for horizontal and vertical cross-sections, with 24 % of vertical uncertainties being below 50 nm.

Finally, we analyze the fitting uncertainty for AMs positions $\Delta\mathbb{M}_i/P$, as it influences the uncertainty of QD position ΔQ_i determination via Supplementary Eq. (11). The medians for $\Delta\mathbb{M}_i/P$ distributions, shown in Supplementary Fig. 7e, are 24.1 nm and 35.1 nm for horizontal and vertical cross-sections. Importantly, 83 % of horizontal and 95 % of vertical cross-section uncertainties $\Delta\mathbb{M}_i/P$ are below 50 nm.

Based on these considerations and the correlation between QD spot brightness and uncertainty of QD position fit $\Delta\mathbb{Q}_i/P$, we estimate that working with only the brightest QD spot in each imaging field (due to the average number of $N_\mathrm{F} \approx 10$ spots detected per field, this amounts to limiting cavity fabrication to 10 % of the registered spots), our fitting accuracy would be $\Delta\mathbb{Q}_i/P < 53.2\,\mathrm{nm}$ and $\Delta\mathbb{Q}_i/P < 37\,\mathrm{nm}$ for horizontal and vertical cross-sections (10th percentiles for the uncertainties distributions). Taking medians for the fitting uncertainty of AMs positions $\Delta\mathbb{M}_i/P$, we find the uncertainty of QD localization $\Delta Q < 80.1\,\mathrm{nm}$, cf. Supplementary Eq. (12). Including the electron beam lithography uncertainty $\Delta C = 40\,\mathrm{nm}$, we find the total uncertainty of cavity placement $\Delta R < 90.3\,\mathrm{nm}$, according to Supplementary Eq. (13) (see Supplementary Table 2). Here, we assume $\left(Q_i - M_{i,l}\right) = 25\,\mu\mathrm{m}$ (center of the field) and for ΔP we take the median value of standard error of mean $\hat{\sigma}_{\bar{P}_i}$ calculated for 84 exemplary μPL maps.

Supplementary Table 2. Uncertainties involved in the determination of the accuracy of cavity positioning ΔR for an example of a quantum dot (QD) with low uncertainty of fitting the QD positions $\Delta\mathbb{Q}_i/P$ – corresponding to a QD being in the 10 % of the brightest spots. $\Delta\mathbb{Q}_i/P$ – 1D fit uncertainty for the QD peak center determination, $\Delta\mathbb{M}_i/P$ – 1D fit uncertainty for the alignment mark (AM) center determination, ΔQ_i – 1D uncertainty of QD localization, ΔQ – uncertainty of QD localization in 2D.

Orientation	$\Delta\mathbb{Q}_i/P$	$\Delta\mathbb{M}_i/P$	$\left(\mathbb{Q}_i - \mathbb{M}_{i,l}\right)\Delta P/P^2$	ΔQ_i	ΔQ	ΔR
Horizontal	≤ 53.2 nm	24.1 nm	15.1 nm	< 61.1 nm	< 80.1 nm	< 90.3 nm
Vertical	≤ 37 nm	35.1 nm		< 53.2 nm		

Supplementary Note 4. OPTICAL SETUPS

Supplementary Fig. 8a presents the setup used for spectroscopy studies of fabricated devices (μPL, extraction efficiency determination, time-resolved μPL), while the above-band autocorrelation histograms are recorded in a setup shown in Supplementary Fig. 8b. The Methods section gives details of the equipment used.

Supplementary Note 5. DETERMINATION OF THE PHOTON EXTRACTION EFFICIENCY

To determine the value of photon extraction efficiency η, we calibrate the optical setup shown in Supplementary Fig. 8a and calculate its efficiency η_Setup as a multiplication of the transmission of all optical elements and of the efficiency of fiber in-coupling and superconducting nanowire single-photon detector (SNSPD) quantum efficiency. We start with reflecting the laser tuned to 1.55 μm off a silver mirror placed in the setup instead of the structure. The signal emitted from the sample passes through the elements enumerated in Supplementary Table 3, given with their measured transmission efficiencies. For SNSPD, we take nominal efficiency. We obtain $\eta_\mathrm{Setup} = (1.10 \pm 0.17)\,\%$, where the uncertainty is calculated by propagating the assumed uncertainties of transmission of the consecutive elements.

Then, we excite the QDs off-resonantly with a pulsed laser diode with $f_\mathrm{rep} = 80\,\mathrm{MHz}$ repetition rate at the saturation power for each QD. We collect the emission with the microscope objective (NA = 0.4) and take the SNSPD count rate n_QD for the most

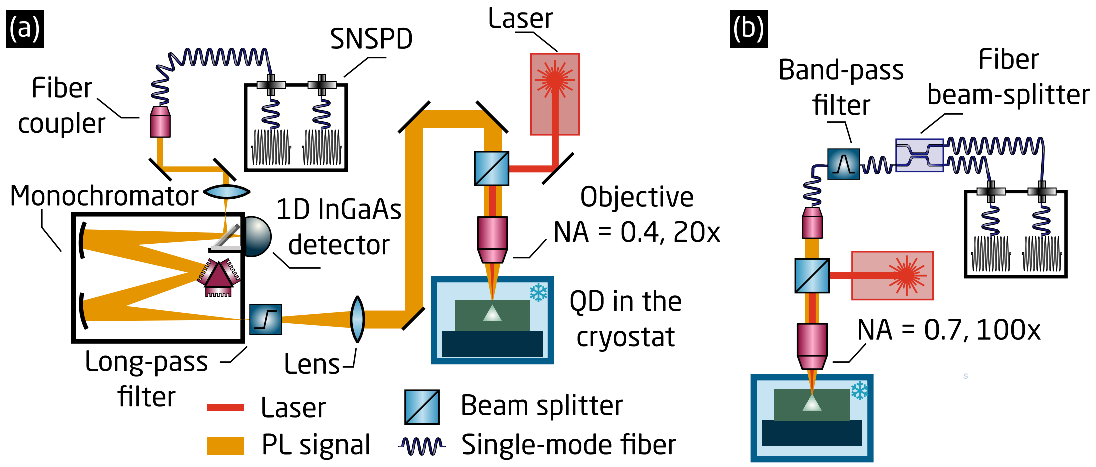

Supplementary Fig. 8. Simplified schemes of the experimental setups used for the device characterization. **a**, the optical setup used for the determination of the photon extraction efficiency and time-resolved microphotoluminescence, **b**, setup used for autocorrelation experiments. SNSPD – superconducting nanowire single-photon detector.

Supplementary Table 3. The transmission of the optical components in the setup used for determination of photon extraction efficiency, as shown in Supplementary Fig. 8a. SNSPD – superconducting nanowire single-photon detector.

Element	Transmission/Efficiency
cryostat window	$(90 \pm 2)\,\%$
microscope objective	$(55 \pm 3)\,\%$
beam splitter	$(38 \pm 2)\,\%$
a set of mirrors	$(85 \pm 5)\,\%$
focusing lens and long-pass filter	$(93 \pm 2)\,\%$
monochromator	$(27 \pm 5)\,\%$
mirrors for signal coupling	$(96 \pm 2)\,\%$
fiber in-coupling	$(41 \pm 10)\,\%$
fibers and their connections	$(80 \pm 10)\,\%$
SNSPD efficiency	$(87 \pm 3)\,\%$
Total setup efficiency η_{Setup}	$(1.10 \pm 0.17)\,\%$

intense QD lines, and correct them by η_{Setup} and f_{rep} according to the formula

$$\eta = \frac{n_{\mathrm{QD}}}{f_{\mathrm{rep}} \times \eta_{\mathrm{Setup}}}. \tag{16}$$

The uncertainty of the photon extraction efficiency for QD-CBG #1 and #2 are calculated by propagating the uncertainties as

$$\Delta\eta = \sqrt{\left(\frac{\Delta n_{\mathrm{QD}}}{\eta_{\mathrm{Setup}}}\right)^2 + \left(\frac{n_{\mathrm{QD}}\Delta\eta_{\mathrm{Setup}}}{\eta_{\mathrm{Setup}}^2}\right)^2}, \tag{17}$$

with $\Delta n_{\mathrm{QD}} = 1000$ and $\Delta\eta_{\mathrm{Setup}} = 0.17\,\%$. This method assumes unity internal quantum efficiency of QDs ($\eta_{\mathrm{int}} = 100\,\%$), so that the QD photon emission rate equals f_{rep}. It is however difficult to determine experimentally the contribution of non-radiative recombination and hence the real value of η_{int}. As a result, a discrepancy arises between the calculated photon extraction efficiency (Fig. 1b) and measured η. The reason for lowered η_{int} (and hence lowered η) can be attributed to the non-radiative recombination channels introduced to the QDs due to structural defects propagating from the InP substrate or defect states at the side walls of the CBG central mesa, which are introduced during dry etching. These defects most likely cause additional exciton energy relaxation channels. The assumption of $\eta_{\mathrm{int}} = 100\,\%$ thus sets a lower limit of η due to a possible overestimation of the total number of photons emitted by the QD.

Supplementary Note 6. SUPPORTING MICROPHOTOLUMINESCENCE DATA FOR QD-CBG DEVICES

A. Quantum dot linewidths

Supplementary Figure 9 presents the histogram of linewidths for all 102 found QDs emitting in the CBGs. We use the FWHM of the fitted Gaussian profiles to describe the linewidth and find that the median linewidth is 0.76 nm, 1st quartile 0.52 nm, and the minimal value 0.14 nm. Additionally, the linewidths for QD-CBGs #1–#3 are shown in Supplementary Table 4.

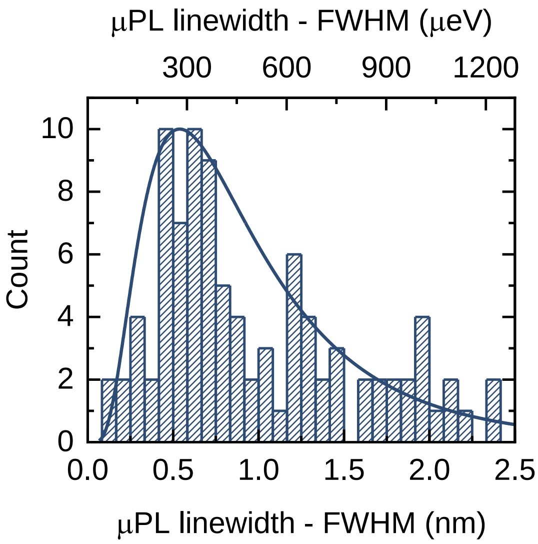

Supplementary Fig. 9. The histogram of linewidths for quantum dots in circular Bragg gratings (full width at half maximum, FWHM, of fitted Gaussian profiles).

Supplementary Table 4. Microphotoluminescence (µPL) linewidths of devices #1–#3.

Device	QD linewidth in µPL
QD-CBG #1	0.47 nm
QD-CBG #2	0.17 nm
QD-CBG #3	0.19 nm

B. Time-resolved microphotoluminescence

Supplementary Figure 10 shows the µPL data supporting the determination of F_P. We focus on an additional QD-CBG #3 with a well-isolated transition line, analogous to QD-CBGs #1 and #2 presented in the article, Fig. 3.

Supplementary Fig. 10a shows a histogram of recorded decay times for the reference QD trion lines. The time-resolved µPL results for eight QDs found in the area outside the fabricated cavities with intense trion lines indicate a considerable scattering of the decay times, with minimal and maximal registered times 1.59 ns and 2.69 ns and the average decay time $\tau_{ref} = 1.99$ ns. The standard deviation of the fitted normal distribution is 0.44 ns and the standard error of mean is 0.16 ns. We take the average decay time $\tau_{ref} = (1.99 \pm 0.16)$ ns to calculate the F_P according to formula $F_P = \tau_{ref}/\tau_{cav}$.

Supplementary Fig. 10b shows the spectrum of QD-CBG #3, and Supplementary Fig. 10c presents time-resolved µPL decay traces in analogy to Fig. 3b of the article, where the same reference time trace is presented. Decay time for QD-CBG #3 is the same as for QD-CBG #2 within the fitting accuracy, $\tau_{\#3} = \tau_{\#2} = (0.53 \pm 0.01)$ ns, what translates to $F_P = (3.75 \pm 0.30)$.

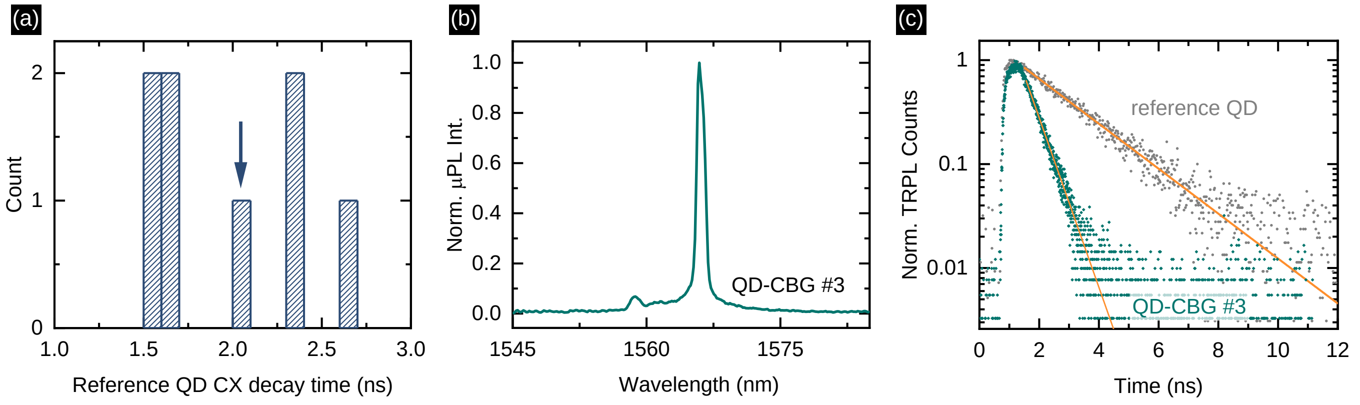

Supplementary Fig. 10. Additional microphotoluminescence (µPL) data. **a**, Histogram of recorded decay times for reference QD trion (CX) lines; arrow points to the quantum dot (QD) shown in Fig. 3b in the article and in panel (c), **b**, µPL spectrum for QD-CBG #3, **c**, time-resolved µPL time traces for QD-CBG #3 and the reference QD.

C. Temperature-dependent µPL

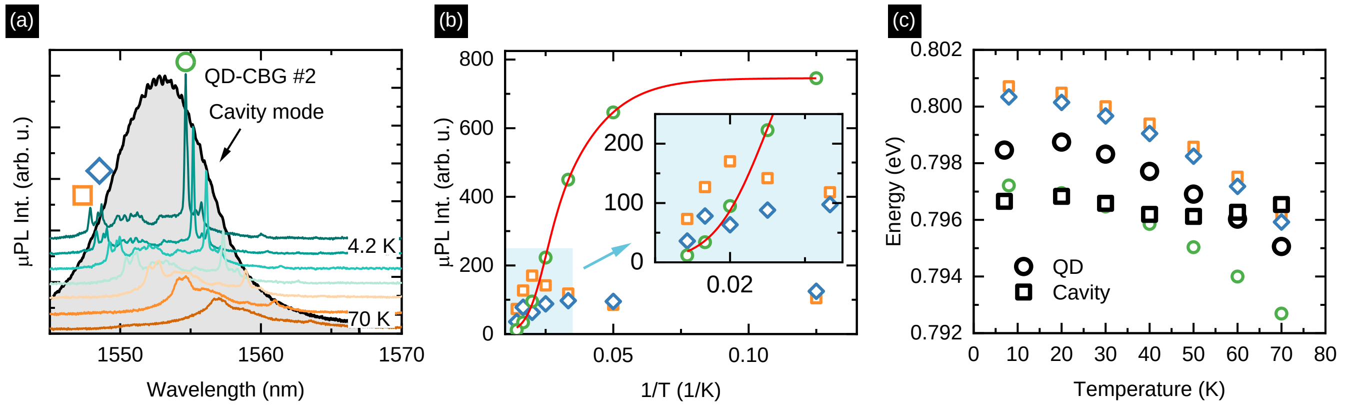

Supplementary Fig. 11. Analysis of the temperature-dependent microphotoluminescence spectra for quantum dot-circular Bragg grating (QD-CBG) device #2. **a**, Temperature-dependent µPL spectra for QD-CBG #2, overlapped with the cavity mode. **b**, Arrhenius plot for the integrated µPL intensity for three QD lines, indicated with the same symbols in panel (a). Inset: close-up of the marked region. **c**, µPL emission energy for the same QD lines. Black symbols stand for another QD-CBG device, with QD and cavity mode energies tracked up to $T = 70\,\mathrm{K}$. The mode crossing characteristic for the weak QD-cavity coupling regime is visible.

We record the temperature-dependent µPL signal for QD-CBG #2 in the temperature range of $T = 4.2\,\mathrm{K}$ to $70\,\mathrm{K}$ under the continuous-wave (CW) excitation and present the stacked spectra in Supplementary Fig. 11a. Three emission lines, marked with rings, can be identified and their emission intensity and energy tracked as the temperature is increased. We plot also the mode profile for reference. Note the $\sim 2\,\mathrm{nm}$ redshift of the most intense line from the central wavelength of the cavity.

The µPL intensities change differently for lines with emission energy lower vs. higher compared to the mode profile. The short-wavelength lines, marked with red and blue rings, are tuned across the mode profile as the temperature raises. Their µPL intensity quench is greatly suppressed, in fact, the intensity of the line marked with a red circle has a maximum at $T = 50\,\mathrm{K}$. The behavior for the long-wavelength line, marked with a green ring, is opposite and its intensity quenches fast.

The temperature-dependent µPL intensity for the most intense line marked with the green ring is fitted with a standard formula assuming two activation processes [11]:

$$I(T) = \frac{I_0}{1 + B_1 \exp(-E_{\mathrm{a},1}/k_\mathrm{B}T) + B_2 \exp(-E_{\mathrm{a},2}/k_\mathrm{B}T)}, \tag{18}$$

where I_0 is the µPL intensity for $T \to 0$, $E_{\mathrm{a},1}$ and $E_{\mathrm{a},2}$ are activation energies, and B_1 and B_2 are relative rates corresponding to the efficiency of involved processes. We achieve the activation energies of $E_{\mathrm{a},1} = (6.9 \pm 0.8)\,\mathrm{meV}$ and $E_{\mathrm{a},2} = (27.9 \pm 3.1)\,\mathrm{meV}$.

We note that $E_{a,2}$ is similar to the activation energy $E_{Ref} = (23.6 \pm 0.8)$ meV found for a CX line from analogous QDs [7]. Accordingly to Ref. [12], this activation energy can be attributed to the charge transfer to higher orbital states, based on the band structure calculations within the 8-band $k \cdot p$ framework. Hence, another mechanism is responsible for the identification of $E_{a,1}$ energy which is absent in the case of high-energy lines (blue and red circles, see Supplementary Fig. 11b). This can potentially be associated with the enhancement of the QD emission rate when the overlap between the line and the mode energy is maximized.

In Supplementary Figure 11c we plot the temperature dependence of the emission energy for lines analyzed in Supplementary Fig. 11b and, additionally, for another investigated QD-CBG device, where the observation of the crossing of the temperature-tuned QD line and the cavity mode under high power off-resonant CW excitation was possible due to the QD energy being higher than the mode energy at $T = 4.2$ K. The temperature-induced evolution of cavity mode energy (black open squares) can be compared with the QD lines, evidencing a weaker temperature dependence, as expected for the cavity mode.

Supplementary Note 7. QUANTUM OPTICS EXPERIMENTS

A. Off-resonant autocorrelation data for QD-CBG #2

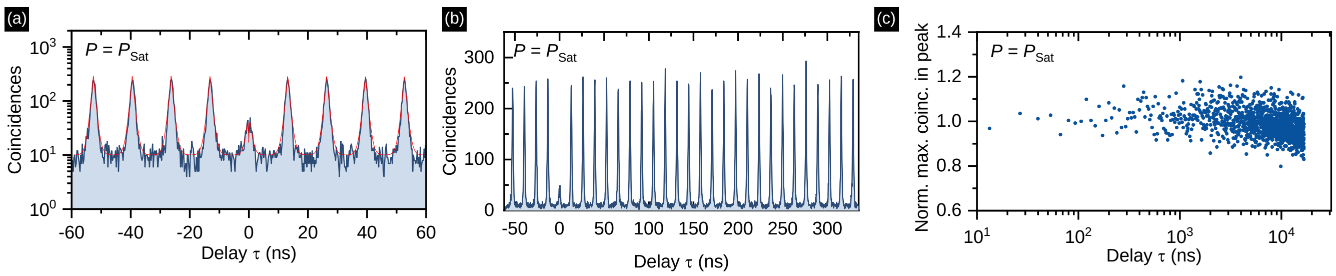

Supplementary Fig. 12. Analysis of the second-order autocorrelation function $g^{(2)}(\tau)$ of the photons emitted from quantum dot-circular Bragg grating (QD-CBG) #2 under pulsed off-resonant excitation for laser excitation power P corresponding to saturation of the trion (CX) line. **a**, **b**, The recorded histogram **c**, Normalized maximum number of coincidences for all 1260 registered peaks for positive delays of histogram shown in (b).

Supplementary Figure 12 shows the autocorrelation histogram recorded under pulsed off-resonant excitation for QD-CBG #2. We do not observe blinking there, as the normalized coincidences in consecutive peaks (maximum number of coincidences without fitting) in the $g^{(2)}(\tau)$ histogram is at a constant level (Supplementary Fig. 12c). We show data for all registered 1260 peaks, i. e. up to $\tau = 16.7\,\mu$s delay. Fluctuation in the data originates in the discretization of the $g^{(2)}(\tau)$ due to finite time binning in the experiment. The blinking would indicate the occupation of a meta-stable QD state [13], originating, e. g., in the emission wavelength fluctuations due to nearby defects [14] or trapped charges [15].

We fit the histograms with the function [16, 17]:

$$C(\tau) = B + A\left[\exp\left(-|\tau|/\tau_{\mathrm{dec}}\right) - \exp\left(-|\tau|/\tau_{\mathrm{cap}}\right)\right] + H\sum_{n\neq 0}\exp\left(-|\tau - n\tau_0|/\tau_{\mathrm{dec}}\right), \tag{19}$$

where B is the level of background coincidences, A is a scaling parameter related to secondary photon emission, $n \neq 0$ is the peak number, τ_0 is the laser pulse period, and H the average height of the peaks at $\tau_n = n\tau_0$. The second-order correlation function $g^{(2)}(\tau)$ is then obtained by normalizing $C(\tau)$ with $(H+B)$.

The time-independent level of background coincidences B originates from the detector dark counts and uncorrelated photons contributing to the registered histograms. Our approach allows taking into account only coincidences caused by the QD signal. We define the purity as the ratio between the QD emission coincidences registered at τ_0 peak (area of this peak) to the average number of coincidences (peak area) registered at $\tau_{n\neq 0}$ peaks. Then, the $g^{(2)}(0)$ value is calculated by first subtracting the background contribution B, integrating the areas under the central peak and under the non-zero peaks, and dividing these two integrals, according to the formula:

$$g^{(2)}(0)_{\mathrm{fit}} = \frac{\int_{-\tau_0/2}^{\tau_0/2} A\left[\exp\left(-|\tau|/\tau_{\mathrm{dec}}\right) - \exp\left(-|\tau|/\tau_{\mathrm{cap}}\right)\right]\mathrm{d}\tau}{\int_{-\tau_0/2}^{\tau_0/2} H\exp\left(-|\tau|/\tau_{\mathrm{dec}}\right)\mathrm{d}\tau}. \tag{20}$$

Supplementary Table 5 summarizes the fit parameters for off-resonant autocorrelation histograms obtained for $0.5 \times P_{sat}$ and P_{sat} excitation power. The uncertainties are determined from the fit uncertainties, and the $g^{(2)}(0)_{fit}$ is calculated from the fit parameters.

Supplementary Table 5. Fitting parameters for off-resonant autocorrelation measurement.

	Off-resonant excitation power	
Fit parameter	$0.5 \times P_{sat}$	P_{sat}
Background contribution, B	(14.4 ± 2.0)	(10.2 ± 0.7)
Scaling of the center peak, A	(54 ± 15)	(67 ± 9)
Height of $\tau_{n \neq 0}$ peaks, H	(187 ± 4)	(286 ± 2)
Laser period, τ_0	(13.14 ± 0.01) ns	(13.15 ± 0.01) ns
Decay time, τ_{dec}	(668 ± 29) ps	(714 ± 25) ps
Recapture time, τ_{cap}	(514 ± 73) ps	(192 ± 42) ps
$g^{(2)}(0)_{fit}$, Supplementary Eq. (20)	(0.05 ± 0.02)	(0.17 ± 0.03)

B. Microphotoluminescence excitation spectroscopy of QD-CBG #2

The two-photon interference experiments were carried out under quasi-resonant excitation conditions. The exact excitation energy was determined based on the microphotoluminescence excitation (µPLE) experiment conducted with a pulsed tunable laser with 5 ps-long pulses and 80 MHz repetition rate. The excitation laser wavelength was varied in the range of 1470-1540 nm at constant average excitation power of 25 µW measured in front of the cryostat window. In the µPLE map, shown in Supplementary Fig. 13a, a clear maximum is visible at the wavelength 1484.2 nm, corresponding to 835.37 meV photon energy which was used for all experiments described in this section. The energy difference of 37.57 meV (see the map cross-section in Supplementary Fig. 13b) coincides reasonably well with the LO phonon energy of InP of 43.4 meV at low temperature [18]. Simultaneously, the measured energy difference is far above the calculated trion p-shell splitting of about ~ 20 meV [12] for QDs very similar in size and chemical composition (P admixture) to the ones investigated here. Therefore, we assume that the applied quasi-resonant excitation of the QD is LO-phonon-assisted.

The comparison of the time-resolved µPL time traces for the trion line in QD-CBG #2 for quasi- and off-resonant excitation, shown in Supplementary Fig. 13c, confirms the accelerated relaxation of the excited state by the reduced decay time under for the quasi-resonant excitation.

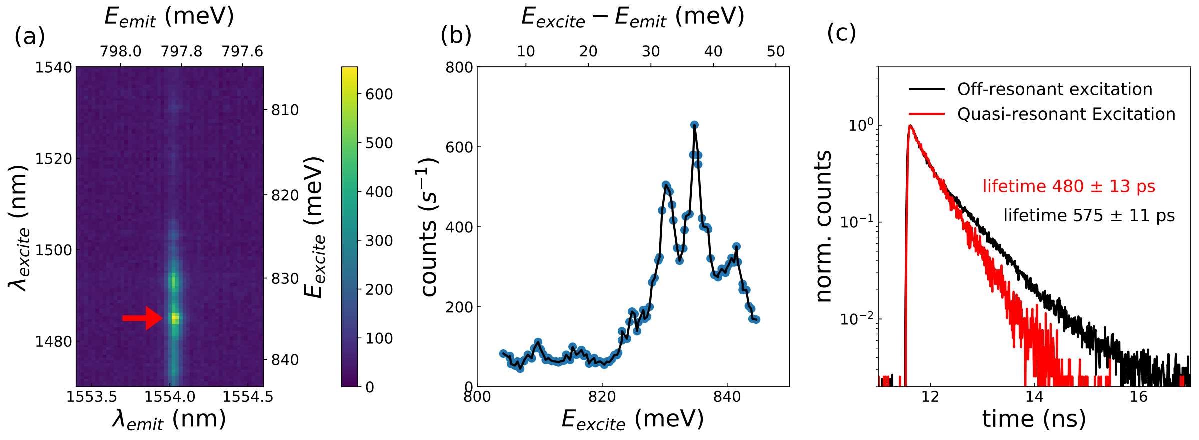

Supplementary Fig. 13. Quasi-resonant excitation of quantum dot-circular Bragg grating (QD-CBG) #2. **a**, Microphotoluminescence excitation map of (QD-CBG) #2. **b**, Cross-section of the map taken at the center of the QD-CBG #2 emission line, revealing the quasi-resonant excitation energy of ~ 0.835 eV used to excite the QD for the indistinguishability measurements. The energy difference corresponds to the detuning of ~ 37 meV from the emission energy. **c**, Time-resolved µPL data for line in QD-CBG #2 for quasi- and off-resonant excitation.

C. Quasi-resonant autocorrelation data for QD-CBG #2

The single-photon purity was verified in autocorrelation measurements taken at various excitation powers to identify the contribution of multi-photon emission and unsuppressed reflected laser light which lead to erroneous coincidences in HOM measurements. The lowest $g^{(2)}(0)$ value was found for the excitation power of $1.5\,\mu$W, and the corresponding autocorrelation data is shown in Fig. 4a of the main text. In this configuration, the autocorrelation histogram was measured directly through the HOM setup to make sure that no reflected laser obscures the HOM experiment. Increasing the excitation power is favored by increasing the signal-to-background ratio as long as the QD is in the linear response regime. On the other hand, increasing the excitation power once the QD emission intensity is saturated results mostly in the increase of the background counts due to the cavity being fed by other sources. They mostly originate in the low-energy tail of the wetting layer emission or radiative defects present in the sample.

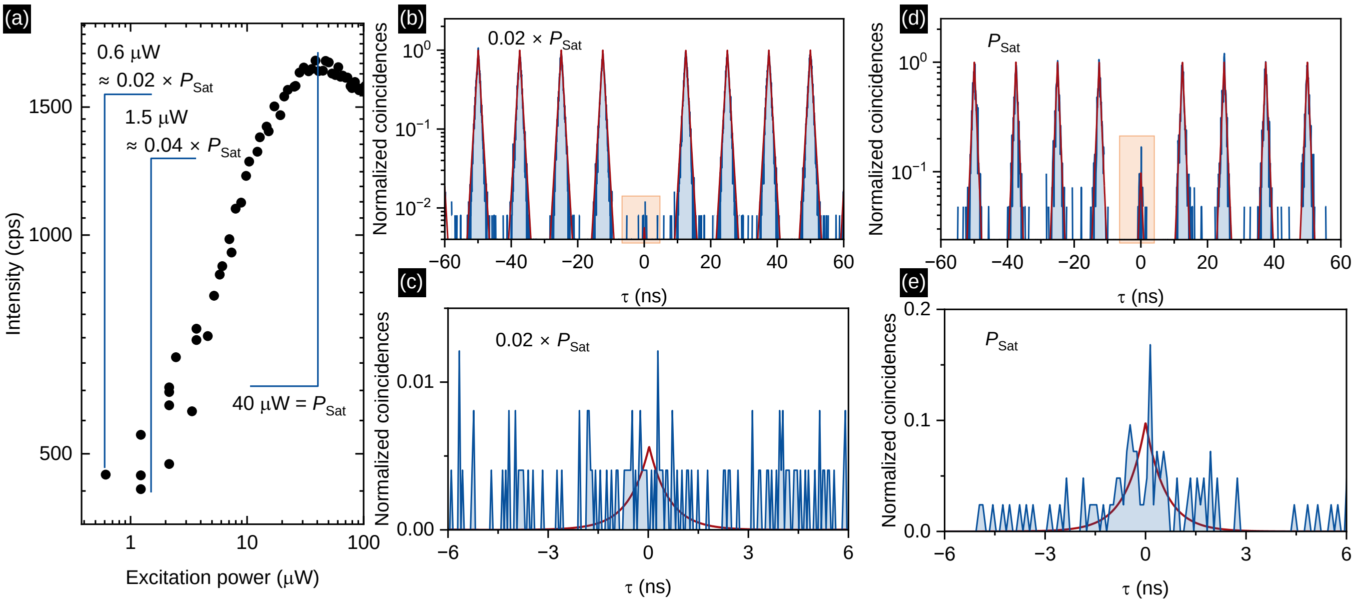

Supplementary Fig. 14. Quasi-resonant autocorrelation data for quantum dot-circular Bragg grating (QD-CBG) #2. **a**, Excitation-power dependent microphotoluminescence (μPL) intensity of the line in QD-CBG #2 under quasi-resonant excitation with the powers chosen for Hong-Ou-Mandel (HOM) measurements marked. **b–e** Autocorrelation histograms for QD-CBG #2 under (b), (c) $P_{\mathrm{exc}} = 0.6\,\mu$W ($0.02 \times P_{\mathrm{sat}}$), (d), (e) $P_{\mathrm{exc}} = 40\,\mu$W ($P_{\mathrm{sat}}$). Orange boxes mark the region that is zoomed in at the bottom.

The low power autocorrelation measurement taken using the HOM configuration leads to an integrated $g^{(2)}(0)_{\mathrm{raw}} = (3.2 \pm 0.6) \times 10^{-3}$ which confirms the suppression of the excitation laser in preparation for the HOM experiment.

To fit the autocorrelation histograms we modify Supplementary Eq. (19) used for fitting off-resonant data, as we do not observe background counts ($B = 0$) and no carrier recapture so that the new formula reads

$$C(\tau) = c \cdot \left(g^{(2)}(0)_{\mathrm{fit}} \exp(-|\tau|/\tau_{\mathrm{dec}}) + \sum_{n \neq 0} \exp(-|\tau - n\tau_0|/\tau_{\mathrm{dec}}) \right), \tag{21}$$

where c is a global normalization factor.

The extracted fit parameters for the quasi-resonantly excited autocorrelation data shown in the main text and in Supplementary Figs. 14b-14c are shown in Supplementary Table 6. Note that in real applications temporal filtering is not always possible which is why we state also the integrated value. For that, we integrate all coincidences in a window of ± 6 ns around each peak in the histogram. Then, we divide the sum of coincidences in the center window by the average of the sums in all side windows, as no blinking is present. The uncertainty is based on the variance of integrated side peak areas.

D. Indistinguishability measurements and data analysis

Supplementary Fig. 15 presents the configuration of the experimental setup applied to record the HOM histograms. The 4 ns excitation delay is compensated on the detection side. A cross-polarization setup suppresses the reflected laser light and a 0.4 nm

Supplementary Table 6. Fitting parameters for quasi-resonant autocorrelation measurement.

	Quasi-resonant excitation power		
Fit parameter	$P_{exc} = 0.6\,\mu W$ ($0.02 \times P_{sat}$), Fig. 14b	$P_{exc} = 1.5\,\mu W$ ($0.04 \times P_{sat}$), Fig. 4a	$P_{exc} = 40\,\mu W$ (P_{sat}), Fig. 14c
Decay time, τ_{dec}	(606 ± 3) ps	(584 ± 3) ps	(591 ± 7) ps
Laser period, τ_0	(12.49 ± 0.01) ns	(12.49 ± 0.01) ns	(12.49 ± 0.01) ns
$g^{(2)}(0)_{fit}$	$(5.6 \pm 5.0) \times 10^{-3}$	$(4.7 \pm 2.6) \times 10^{-3}$	$(9.81 \pm 1.94) \times 10^{-2}$
$g^{(2)}(0)_{raw}$	$(4.2 \pm 0.2) \times 10^{-3}$	$(3.2 \pm 0.6) \times 10^{-3}$	$(8.75 \pm 4.88) \times 10^{-2}$

fiber bandpass spectrally filters the emission. The HOM setup consists of a 50:50 free-space beam splitter and a 50:50 fiber beam splitter in which the interference takes place. The fiber in-coupling can be translated for optimization of the temporal matching and the polarization is set in free space via waveplates and confirmed with a polarimeter.

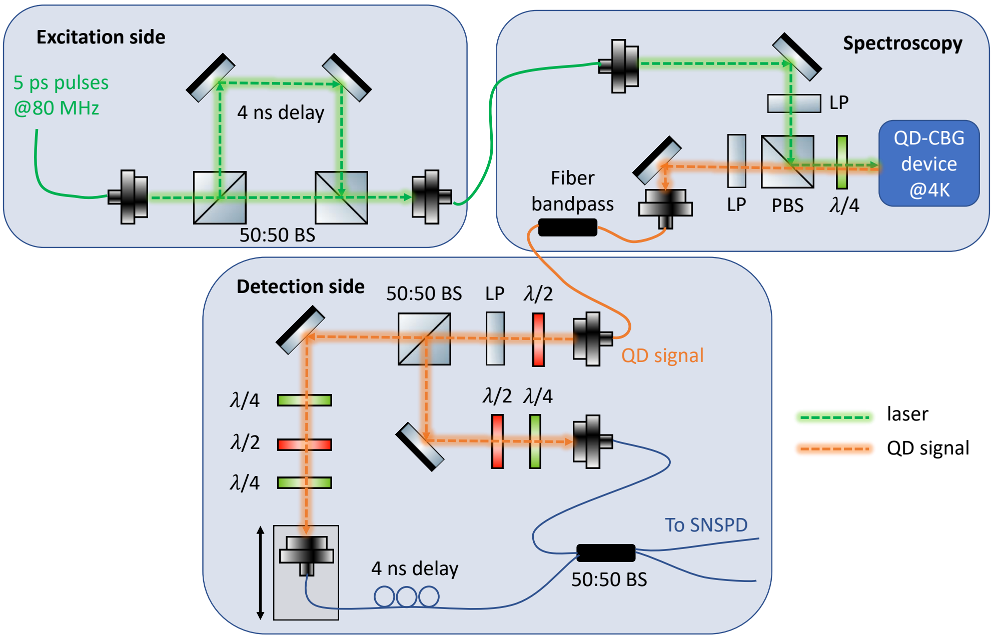

Supplementary Fig. 15. The Hong-Ou-Mandel (HOM) experimental setup. BS – beam splitter, $\lambda/2$, $\lambda/4$ – half and quarter wave-plate, SNSPD – superconducting nanowire single-photon detector.

The indistinguishability measurements were performed with a 4 ns delay. To do so, the train of laser pulses arriving every 12.5 ns was split on the excitation side into two pulses separated by a delay of 4 ns which was compensated on the detection side, to interfere subsequently emitted photons in a fiber beam splitter. The laser was tuned to excite the QD-CBG quasi-resonantly for three excitation powers, $P_{exc} = 0.6\,\mu W$ ($0.02 \times P_{sat}$), $P_{exc} = 1.5\,\mu W$ ($0.04 \times P_{sat}$), and $P_{exc} = 40\,\mu W$ (P_{sat}).

The obtained histograms are composed of a characteristic pattern of 5 peaks repeated every 12.5 ns corresponding to the 80 MHz repetition rate of the excitation laser. They are related to coincidences resulting from consecutive photons taking different paths in the imbalanced Mach-Zehnder interferometer (MZI). There are 5 possible final delay combinations leading to the observed pattern [19]. For the Poissonian statistics of the emission, the intensity ratio of the 5 non-central peaks is expected to be 1:4:6:4:1, whereas for the center peak (produced by coincidences originating in the pair of laser pulses separated by 4 ns), indistinguishable single photons produce the combination of coincidences 1:2:0:2:1 in contrast to 1:2:2:2:1 for completely distinguishable photons.

The figure of merit for the photon indistinguishability can be extracted from HOM measurements in different ways. Typically, the amount of coincidences in the case of expected indistinguishability is compared to the number of coincidences for expected maximum distinguishability, either at cross-polarized interference or from different laser pulses (photons that have not interfered). As the second approach with side peaks is more susceptible to blinking and imperfect setups, we determine the indistinguishability from the comparison of co- and cross-polarized HOM measurements and extract the visibility as the ratio of the central peak areas

via

$$V = 1 - A_{\mathrm{Co}}/A_{\mathrm{Cross}}, \tag{22}$$

while we still mention the values obtained from the side peak method for completeness at the end of this section. In order to extract also other physical quantities and to compensate for statistical fluctuations the data is fitted according to the model described below and the areas $A_{\mathrm{Co}} = A_3$ for the co-polarized case and $A_{\mathrm{Cross}} = A_3$ for the cross-polarized case are obtained.

To properly fit the entire HOM coincidence histograms one has to keep in mind that the areas of the ± 4 ns side peaks belonging to a laser pulse at the time delay $\tau = 0$ ns already overlap with the areas from the side peaks originating from the ± 8 ns laser excitation at $\tau = \pm 12.5$ ns (as they are present in the histogram at ± 4.5 ns), see Supplementary Fig. 16. Consequently, the individual contributions can be extracted by fitting the data by the sum of all contributions according to the formula. For the HOM histogram recorded for the co-polarized case, the formula reads

$$\begin{aligned} C_{\mathrm{HOM,Co}}(\tau,[\tau_1,T_2,\Delta t,\tau_0,\mathbf{A},\mathbf{B}]) &= A_3\exp(-|\tau|/\tau_1)\left(1 - V_{\mathrm{PS}}\cdot e^{-|\tau|/T_2}\right) + \sum_{\mathrm{i}=\{1,2,4,5\}} A_i\exp(-|\tau+\Delta t_i|/\tau_1) \\ &+ \sum_{n=-10,n\neq 0}^{10}\left[\sum_{\mathrm{i}=\{1,2,3,4,5\}} B_i\exp(-|\tau+\Delta t_i+n\cdot\tau_0|/\tau_1)\right], \end{aligned} \tag{23}$$

and for the cross-polarized data

$$\begin{aligned} C_{\mathrm{HOM,Cross}}(\tau,[\tau_1,T_2,\Delta t,\tau_0,\mathbf{A},\mathbf{B}]) &= A_3\exp(-|\tau|/\tau_1) + \sum_{\mathrm{i}=\{1,2,4,5\}} A_i\exp(-|\tau+\Delta t_i|/\tau_1) \\ &+ \sum_{n=-10,n\neq 0}^{10}\left[\sum_{\mathrm{i}=\{1,2,3,4,5\}} B_i\exp(-|\tau+\Delta t_i+n\cdot\tau_0|/\tau_1)\right]. \end{aligned} \tag{24}$$

These formulas for the normalized coincidences as a function of the detection time difference τ includes the 4 ns delay between two interfering photons $\Delta t_{1-5} = \{-8,-4,0,4,8\}$ ns, the 12.5 ns initial laser pulse delay τ_0, the photoluminescence decay time τ_1, the coherence time T_2, the post-selected visibility V_{PS}, the respective peak heights of the center 5-peak-structure A_{1-5}, and the averaged peak heights of all peaks at higher delays B_{1-5}. The two fits for co- and cross-polarized cases differ only by the existence of the volcano-shaped dip in the central peak of the co-polarized data, being the fingerprint of the two-photon interference. The post-selected visibility is the value one obtains also when comparing the center peak contribution of the co- and the cross-case at $\tau = 0$ as then

$$V_{\mathrm{PS}} = 1 - \frac{C_{\mathrm{HOM,Co}}(\tau=0)}{C_{\mathrm{HOM,Cross}}(\tau=0)} = 1 - \frac{A_3(1-V_{\mathrm{PS}})}{A_3}. \tag{25}$$

The simulated histograms for our experimental parameters expected from this model are shown in Supplementary Fig. 16 top and bottom for perfectly indistinguishable and distinguishable photons, respectively.

While the fitted histogram for $0.04\times P_{\mathrm{sat}}$ is presented in the article, Fig. 4b, the fitted histograms for $0.02\times P_{\mathrm{sat}}$ and P_{sat} excitation powers are shown in Supplementary Fig. 17 and the extracted parameters in Supplementary Table 7. All fits were done on the unbinned raw data without correcting for finite $g^{(2)}(0)$, as the obtained $g^{(2)}(0)$ values are small. Additionally, the fits do not include corrections for the detector time response or subtracting a fixed background. The uncertainties are determined from the fit errors and propagated whenever a quantity was calculated from the fit parameters.

One can clearly see from the graph that the photon indistinguishability is reduced at higher power, as evidenced by the larger central peak area. That is confirmed also by the extracted visibilities of (22.1 ± 8.9) %, (19.3 ± 2.6) % and (11.3 ± 2.3) % for low to high power. That is partly due to a worse single-photon purity at P_{sat} (see Supplementary Fig. 14c) but mainly due to a reduced coherence, as indicated by the narrower central dip at higher excitation power. We find that the coherence time T_2 is reduced from (176 ± 9) ps over (103 ± 13) ps to (74 ± 6) ps with the increasing excitation power. The variation between the extracted post-selected values of $V_{\mathrm{PS}} = (80\pm 13)$ %, (99 ± 6) % and (84 ± 3) % for different excitation powers results from the finite temporal resolution of our setup (57 ps FWHM jitter that is especially critical when resolving short coherence times), limited statistics due to reduced count rates at lower excitation powers, also evidenced by the larger error and the increased multi-photon contributions at higher power. Correcting for the $g^{(2)}(0)$ increase at higher powers, the extracted post-selected visibility values agree within the standard errors. However, to be in line with future real-world applications, we state the uncorrected values. The larger uncertainties for the fit parameters for the HOM histogram recorded at $0.02\times P_{\mathrm{sat}}$ are caused by the lower gathered statistics, as also indicated by the larger mean fit residuals (MFR) for the fit of the co-polarized case (0.0812 vs. 0.0280). Note that for the same reason, the deviation from the expected peak ratios is also larger in this case. The remaining deviations from the expected 5-peak-ratios (**A**, **B**) can also be caused by unequal transmissions in the two arms of the MZIs.

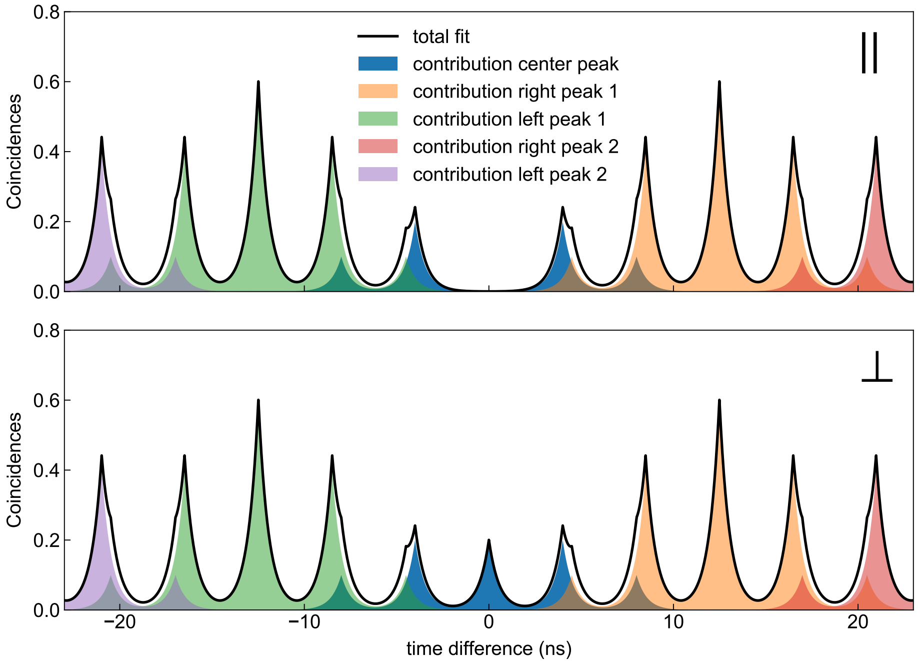

Supplementary Fig. 16. Expected Hong-Ou-Mandel (HOM) histograms of coincidences with individual peak contributions to the five peak patterns formed by 4 ns delay and laser repetition of $\tau_0 = 12.5\,\mathrm{ns}$, **a**, for perfectly indistinguishable photons, and **b**, for maximally distinguishable photons. The black lines represent the fits according to Supplementary Eq. (23)hom-pulsed-Cross that include all contributions, plotted for $\tau_1 = 550\,\mathrm{ps}$ lifetime.

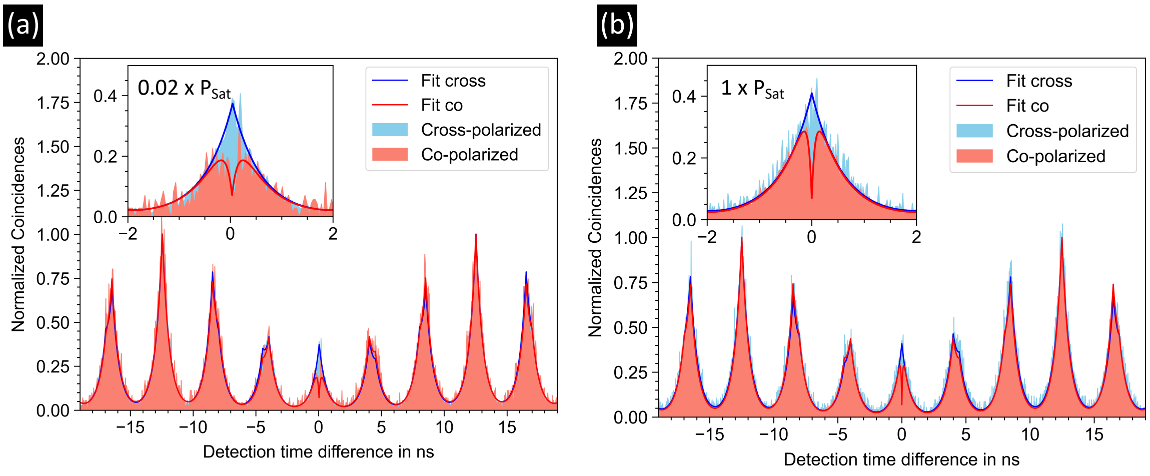

Supplementary Fig. 17. Comparison of Hong-Ou-Mandel (HOM) histograms taken at **a**, $0.02 \times P_{\mathrm{sat}}$ and **b**, P_{sat} excitation power. HOM measurement recorded for $0.02 \times P_{\mathrm{sat}}$ shows higher indistinguishability and longer coherence time than the one taken at P_{sat}, see Supplementary Table 7.

As mentioned at the beginning of this section, in addition to comparing the co- and cross-polarized central peak areas, the indistinguishability can also be extracted by comparing the areas of the central to the outer peaks for only the co-polarized data. This analysis of the low-power HOM histogram is presented in Supplementary Fig. 18. Evaluating the visibility from the extracted center A_3 and side peak areas A_2, A_4, and calculating the visibility as $V_{\mathrm{side\,peaks}} = 1 - 2A_3/(A_2 + A_4)$ leads to $(15.9 \pm 9.9)\,\%$ for the low excitation power $(0.02 \times P_{\mathrm{sat}})$, which agrees with the visibility result reported above obtained from the co-cross-comparison. The uncertainty is however larger, as the fitting relies on successfully separating the overlapping peak contributions.

Importantly, the obtained visibilities and the post-selected values compare favorably with the reports for GaAs-based QDs emitting at C-band [20–22]. For planar QDs, a visibility of $(14.4 \pm 1.5)\,\%$ has been reported under pulsed resonant excitation [21]

Supplementary Table 7. Extracted fitting parameters for recorded Hong-Ou-Mandel (HOM) histograms. MFR – mean fit residuals, TPI – two-photon interference, pol – polarized.

Fit parameter	Quasi-resonant excitation power		
	$0.02\times P_{sat}$, shown in Fig. 17a	$0.04\times P_{sat}$, shown in Fig. 4c	P_{sat}, shown in Fig. 17b
Co-pol areas **A**, **B**	$\begin{pmatrix}1.00\pm0.11\\2.08\pm0.11\\1.77\pm0.21\\2.14\pm0.11\\1.38\pm0.11\end{pmatrix},\begin{pmatrix}1.00\pm0.06\\4.23\pm0.05\\6.66\pm0.04\\4.55\pm0.05\\1.27\pm0.06\end{pmatrix}$	$\begin{pmatrix}1.00\pm0.04\\1.84\pm0.04\\1.68\pm0.05\\1.95\pm0.04\\1.03\pm0.04\end{pmatrix},\begin{pmatrix}1.00\pm0.02\\3.76\pm0.02\\5.69\pm0.02\\3.75\pm0.02\\1.03\pm0.02\end{pmatrix}$	$\begin{pmatrix}1.00\pm0.02\\1.96\pm0.02\\2.02\pm0.03\\1.92\pm0.02\\0.95\pm0.02\end{pmatrix},\begin{pmatrix}1.00\pm0.01\\3.78\pm0.01\\5.68\pm0.01\\3.75\pm0.01\\0.98\pm0.01\end{pmatrix}$
Cross-pol area A_3,	2.28 ± 0.04	2.08 ± 0.04	2.27 ± 0.06
Lifetime, τ_1	(559 ± 4) ps	(553 ± 2) ps	(563 ± 7) ps
Coherence time, T_2	(176 ± 9) ps	(103 ± 13) ps	(74 ± 6) ps
V_{PS}	(80 ± 13) %	(99 ± 6) %	(84 ± 3) %
MFR, Co-pol fit	0.0812	0.0286	0.0158
MFR, Cross-pol fit	0.0280	0.0280	0.0526
TPI visibility, V	(22.1 ± 8.9) %	(19.3 ± 2.6) %	(11.3 ± 2.3) %

while the raw visibility value of (71 ± 15) % was obtained under two-photon-resonant CW excitation [20]. Values obtained under CW excitation can be related to the post-selected values determined from pulsed excitation. For QDs placed non-deterministically in CBGs, the only reported visibility so far is (8.1 ± 3.4) % and the post-selected on the order of 60 % [22].

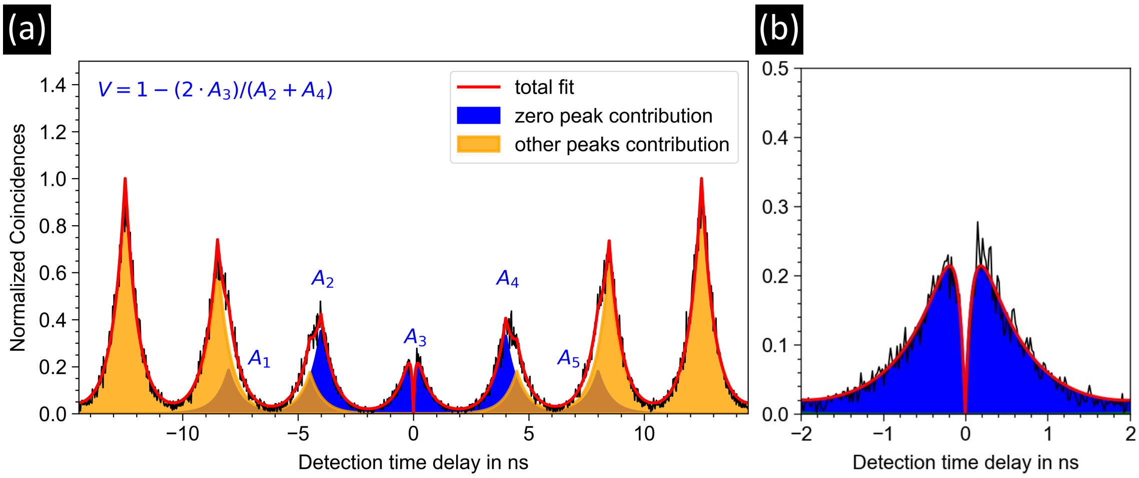

Supplementary Fig. 18. Data analysis of the low-power Hong-Ou-Mandel (HOM) histogram. **a**, Individual peak contributions extracted as part of the total fit according to Supplementary Eq. (23) for the lowest power HOM measurement ($0.02\times P_{sat}$). **b**, Close-up of the central peak.

E. Coherence measurements

To access the coherence time T_2 directly, we performed measurements using a Michelson interferometer (MI) under above-barrier CW excitation (980 nm) of QDs integrated into CBGs and planar sample regions. To do so, we employed an all-fiber MI shown in Supplementary Fig. 19a, where the inset shows a typical interference scan. While the resulting T_2 values are not directly comparable to the values extracted from the HOM experiments, they provide lower bounds on the coherence time (as above-barrier CW excitation results typically in substantially enhanced decoherence effects), and can be used to compare different QD devices. The highest coherence time observed in the MI measurements was obtained from the QD-CBG #2 from the main text to be (62 ± 3) ps (c.f. Supplementary Fig. 19b), extracted from double-exponential fits. Note the artifacts, which

are caused by temporary spectral jumps (telegraphic noise), as also discussed in the main text as one of the factors limiting the indistinguishability. The extracted T_2 value is in line with coherence times of ~ 50 ps reported in the literature for InAs/InP QDs grown in the Stranski-Krastanov mode [23].

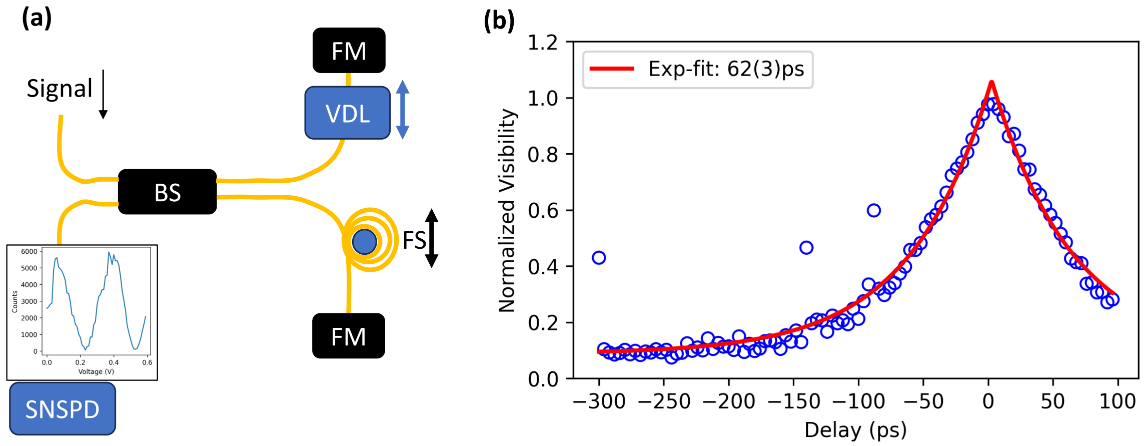

Supplementary Fig. 19. **a**, Schematic of the all-fiber Michelson interferometer for 1550 nm. The single photon signal is split in a fiber beam splitter (BS) and back-reflected at two Faraday mirrors (FM), while the coarse delay on one arm is controlled with a variable optical delay line (VDL) and the fine scan of the relative path difference is done via a piezo fiber stretcher (FS). The counts after the interference are detected on a superconducting nanowire single photon detector (SNSPD). Inset: Example of the FS scan for 0 ps delay indicating constructive and destructive interference. **b**, Extracted visibility for different delay positions allows the determination of the T_2 time using an exponential fit. This is data under 980 nm CW excitation for the QD-CBG #2 from the main text.

To gain further insight, we have measured the T_2 time for the QD-CBG #2 from the manuscript for different excitation powers (Supplementary Fig. 20a) as well as for different temperatures (Supplementary Fig. 20b). As expected, the T_2 time drops when the temperature or the excitation power is increased. The slight increase in coherence when temperature increases might be explained by the fact that the trion transition occasionally showed a random telegraphic noise. Here, the different states may be associated with different coherence times, as confirmed by repeated measurements under the same conditions. The sudden spectral jumps during a Michelson scan yield sudden changes in signal rate, which leads to outliers for the extracted interference contrast (c.f., four data points in Supplementary Fig. 20b).

While the strong dependence of the coherence on excitation power makes a direct comparison between QDs in the planar state and in CBGs difficult, we chose a compromise of evaluating the coherence for 3 exemplary (one high coherence, one medium, and one low) planar and CBG-integrated QDs, respectively. The results are compared in Supplementary Fig. 21. As the planar QDs do not couple to the excitation laser efficiently, more power was required to get a sufficient signal, yielding an overall reduced coherence. Also, due to the smaller count rate, the maximum interference contrast is not well resolved. We do find, however, a spread of measured maximum coherence times between 6-30 ps for the set of planar QDs investigated (Supplementary Fig. 21a).

For the QDs deterministically integrated into CBG structures, a lower excitation power is possible. Even though only a small number of QDs were investigated, the spread of coherence times from 18-62 ps indicates at least no deterioration of the coherence by integrating it into CBG structures. If the integration yields a significant Purcell enhancement, thus reducing the T_1 time, while not strongly reducing the T_2 time, as it seems to be the case here, one can conclude that the CBG cavity brings the emission closer to the Fourier limit of $T_1 = 2 \times T_2$.

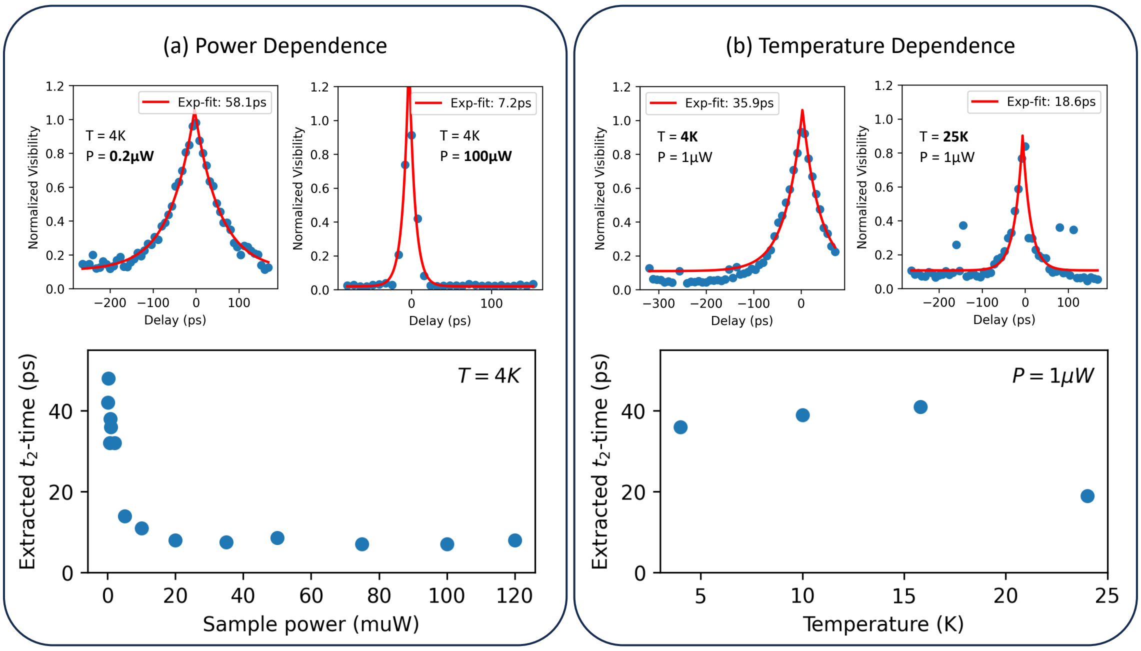

Supplementary Fig. 20. **a**, Measured coherence of the QD-CBG device as a function of excitation power shows coherence drop when increasing power. **b**, Coherence as a function of temperature shows a slight increase, potentially due to a spectral jump into a more coherent state, before decreasing as expected.

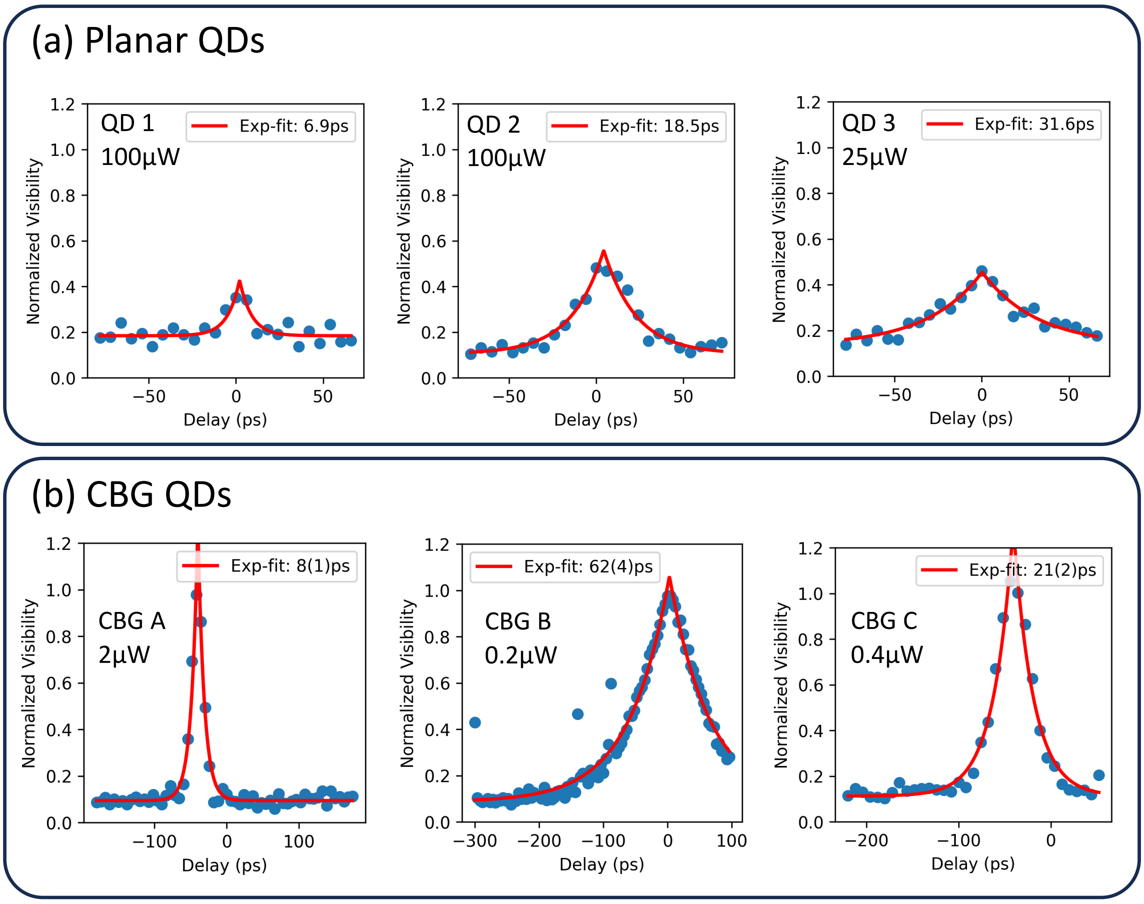

Supplementary Fig. 21. Comparison of coherence measurements results. **a**, Data for the selection of planar QDs, **b**, QDs deterministically integrated into CBG structures. While the exact coherence time depends on power, the general trend is that the T_2 time is, on average, not reduced when integrating the QD into a photonic structure.

[1] U. M. Gür, S. Arslanagić, M. Mattes, and N. Gregersen, Open-geometry modal method based on transverse electric and transverse magnetic mode expansion for orthogonal curvilinear coordinates, Phys. Rev. E **103**, 033301 (2021).
[2] L. Novotny and B. Hecht, *Principles of Nano-Optics*, 2nd ed. (Cambridge University Press, 2012).
[3] A. V. Lavrinenko, J. Lægsgaard, and N. Gregersen, *Numerical Methods in Photonics* (CRC PR INC, 2014).
[4] L. Li, Formulation and comparison of two recursive matrix algorithms for modeling layered diffraction gratings, J. Opt. Soc. Amer. A **13**, 1024 (1996).
[5] C. A. Balanis, *Antenna Theory: Analysis and Design* (Wiley John + Sons, 2016).
[6] B. Yao, R. Su, Y. Wei, Z. Liu, T. Zhao, and J. Liu, Design for Hybrid Circular Bragg Gratings for a Highly Efficient Quantum-Dot Single-Photon Source, J. Korean Phys. Soc. **73**, 1502 (2018).
[7] P. Holewa, A. Sakanas, U. M. Gür, P. Mrowiński, A. Huck, B.-Y. Wang, A. Musiał, K. Yvind, N. Gregersen, M. Syperek, and E. Semenova, Bright Quantum Dot Single-Photon Emitters at Telecom Bands Heterogeneously Integrated on Si, ACS Photonics **9**, 2273 (2022).
[8] A. Sakanas, E. Semenova, L. Ottaviano, J. Mørk, and K. Yvind, Comparison of processing-induced deformations of InP bonded to Si determined by e-beam metrology: Direct vs. adhesive bonding, Microelectron. Eng. **214**, 93 (2019).
[9] B. Zhang, J. Zerubia, and J.-C. Olivo-Marin, Gaussian approximations of fluorescence microscope point-spread function models, Appl. Opt. **46**, 1819 (2007).
[10] J. Liu, M. I. Davanço, L. Sapienza, K. Konthasinghe, J. V. D. M. Cardoso, J. D. Song, A. Badolato, and K. Srinivasan, Cryogenic photoluminescence imaging system for nanoscale positioning of single quantum emitters, Rev. Sci. Instrum. **88**, 023116 (2017).
[11] J. D. Lambkin, D. J. Dunstan, K. P. Homewood, L. K. Howard, and M. T. Emeny, Thermal quenching of the photoluminescence of InGaAs/GaAs and InGaAs/AlGaAs strained-layer quantum wells, Appl. Phys. Lett. **57**, 1986 (1990).
[12] P. Holewa, M. Gawełczyk, C. Ciostek, P. Wyborski, S. Kadkhodazadeh, E. Semenova, and M. Syperek, Optical and electronic properties of low-density InAs/InP quantum-dot-like structures designed for single-photon emitters at telecom wavelengths, Phys. Rev. B **101**, 195304 (2020).
[13] C. Santori, M. Pelton, G. Solomon, Y. Dale, and Y. Yamamoto, Triggered single photons from a quantum dot, Phys. Rev. Lett. **86**, 1502 (2001).
[14] M.-E. Pistol, P. Castrillo, D. Hessman, J. A. Prieto, and L. Samuelson, Random telegraph noise in photoluminescence from individual self-assembled quantum dots, Phys. Rev. B **59**, 10725 (1999).
[15] H. Robinson and B. Goldberg, Light-induced spectral diffusion in single self-assembled quantum dots, Phys. Rev. B **61**, R5086 (2000).
[16] P. A. Dalgarno, J. McFarlane, D. Brunner, R. W. Lambert, B. D. Gerardot, R. J. Warburton, K. Karrai, A. Badolato, and P. M. Petroff, Hole recapture limited single photon generation from a single n-type charge-tunable quantum dot, Appl. Phys. Lett. **92**, 193103 (2008).
[17] T. Miyazawa, K. Takemoto, Y. Nambu, S. Miki, T. Yamashita, H. Terai, M. Fujiwara, M. Sasaki, Y. Sakuma, M. Takatsu, T. Yamamoto, and Y. Arakawa, Single-photon emission at 1.5 µm from an InAs/InP quantum dot with highly suppressed multi-photon emission probabilities, Appl. Phys. Lett. **109**, 132106 (2016).
[18] G. Irmer, M. Wenzel, and J. Monecke, The temperature dependence of the LO(T) and TO(T) phonons in GaAs and InP, Phys. Status Solidi B **195**, 85 (1996).
[19] A. Thoma, P. Schnauber, M. Gschrey, M. Seifried, J. Wolters, J. H. Schulze, A. Strittmatter, S. Rodt, A. Carmele, A. Knorr, T. Heindel, and S. Reitzenstein, Exploring dephasing of a solid-state quantum emitter via time- and temperature-dependent Hong-Ou-Mandel experiments, Phys. Rev. Lett. **116**, 1 (2016).
[20] C. Nawrath, F. Olbrich, M. Paul, S. L. Portalupi, M. Jetter, and P. Michler, Coherence and indistinguishability of highly pure single photons from non-resonantly and resonantly excited telecom C-band quantum dots, Appl. Phys. Lett. **115**, 023103 (2019).
[21] C. Nawrath, H. Vural, J. Fischer, R. Schaber, S. L. Portalupi, M. Jetter, and P. Michler, Resonance fluorescence of single In(Ga)As quantum dots emitting in the telecom C-band, Appl. Phys. Lett. **118**, 244002 (2021).
[22] C. Nawrath, R. Joos, S. Kolatschek, S. Bauer, P. Pruy, F. Hornung, J. Fischer, J. Huang, P. Vijayan, R. Sittig, M. Jetter, S. L. Portalupi, and P. Michler, Bright Source of Purcell-Enhanced, Triggered, Single Photons in the Telecom C-Band, Adv. Quantum Technol. **6**, 2300111 (2023).
[23] M. Anderson, T. Müller, J. Skiba-Szymanska, A. B. Krysa, J. Huwer, R. M. Stevenson, J. Heffernan, D. A. Ritchie, and A. J. Shields, Coherence in single photon emission from droplet epitaxy and Stranski–Krastanov quantum dots in the telecom C-band, Appl. Phys. Lett. **118**, 014003 (2021).
